# A comparative UPLC-orbitrap-MS-based metabolite profiling of three *Pelargonium* species cultivated in Egypt

**DOI:** 10.1038/s41598-024-72153-0

**Published:** 2024-10-01

**Authors:** Rana M. Merghany, Mohamed A. Salem, Shahira M. Ezzat, Sherifa F. A. Moustafa, Salma A. El-Sawi, Meselhy R. Meselhy

**Affiliations:** 1 Department of Pharmacognosy, National Research Centre, 33 El Buhouth St., Cairo, 12622 Egypt; 2 Department of Pharmacognosy and Natural Products, Faculty of Pharmacy, Menoufia University, Gamal Abd El Nasr St., Shibîn el Kôm, 32511 Menoufia Egypt; 3 Department of Pharmacognosy, Faculty of Pharmacy, Cairo University, Kasr El-Aini Street, Cairo, 11562 Egypt; 4 Department of Pharmacognosy, Faculty of Pharmacy, October University for Modern Sciences and Arts (MSA), Giza, 12451 Egypt; 5 Present Address: The BioActives Lab, Biological and Environment Science and Engineering (BESE), King Abdullah University of Science and Technology (KAUST), Thuwal, Saudi Arabia

**Keywords:** *P. graveolens*, *P. fragrans*, *P. denticulatum*, Metabolomics, LC–MS/MS, Molecular networking, Analytical chemistry, Cheminformatics, Secondary metabolism

## Abstract

Several *Pelargonium* species are cultivated mainly to produce essential oils used in perfume industry and for ornamental purposes. Although the chemical composition and biological activities of their essential oils were extensively investigated, there is limited information about the chemical composition of their non-volatile constituents. In this study, we report an Ultra-Performance Liquid Chromatography-Mass Spectrometry (UPLC-MS)-based metabolomics approach for the annotation and analysis of various metabolites in three species; *P. graveolens, P. denticulatum,* and *P. fragrans* utilizing The Global Natural Product Social Molecular Networking (GNPS) and multivariate data analyses for clustering of the metabolites. A total of 154 metabolites belonging to different classes were annotated. The three species are good sources of coumarins, benzoic acid derivatives, organic acids, fatty acids, and phospholipids. However, the highest level of flavonols (mono- and di-*O*-glycosides) and cinnamic acid derivatives was found in *P. graveolens* and *P. denticulatum*, whereas tannins and flavone *C*-glycosides were abundant in *P. fragrans*. The metabolic profiles clarified here provide comprehensive information on the non-volatile constituents of the three *Pelargonium* species and can be employed for their authentication and possible therapeutic applications.

## Introduction

Genus *Pelargonium* is a member of the family Geraniaceae and comprises approximately 280 species^1^. *Pelargonium* plants are noted for their scented leaves which are often used for the production of high-quality essential oils known as “geranium oils” with wide applications in perfumery, cosmetics, and aromatherapy^1^. Research on *Pelargonium* species focused mainly on the chemical composition of essential oils^2,3^. However, the leaves are also rich in non-volatile constituents; coumarins, ellagitannins, proanthocyanidins, and flavonoids with their glycosides and methyl ethers^4^.

Further, the leaves have a long history of traditional use in Africa for the treatment of various ailments such as diarrhea, dysentery, fever, respiratory tract infections, gastroenteritis, hemorrhage, kidney and bladder disorders^5^, as well as anxiety and depression^6^. Furthermore, a traditional herbal medicine (Umckaloabo) prepared from the roots of two *Pelargonium* species clinically proved to treat acute bronchitis and symptoms of upper respiratory tract infections^7^. Besides, the roots of *P. sidoides* demonstrated in situ inhibition of the adhesion of *Helicobacter pylori* to human stomach^8^.

Three *Pelargonium* species; *P. graveolens, P. denticulatum,* and *P. fragrans* were selected for this study. These species are native to South Africa and cultivated worldwide for ornamental and commercial purposes.

*Pelargonium graveolens* is extensively cultivated in Egypt for its rose-scented geranium oil which is mainly composed of citronellol and geraniol^9,10^. Egypt comes as the second largest producer of geranium oil after China and exported more than 250 tons of the oil in 2020. Though there is a high demand for Egyptian geranium oil, there is a growing production of synthetic geranium oil^11^. However, variations or oil adulteration can be detected by Gas Chromatography-Mass Spectrometry (GC–MS)^12^. On the other hand, the rose-scented geranium oil showed a broad spectrum of biological activities such as antioxidant, anti-inflammatory, antimicrobial^3^, antidepressant^13–16^, and attenuation of memory impairment and neuronal death^17,18^. However, only few studies have been conducted regarding the biological activities of its crude extracts^19–23^.

*Pelargonium denticulatum* is a toothed-leaved *Pelargonium* species of exceptional delicacy and balsam-scented leaves^24^. Its essential oil mainly contains limonene, *p*-cymene, hexenyl butyrate, and sesquiterpenes as it proved to possess potent antibacterial activity^25^. However, no information was reported about the chemical composition or biological activities of the organic extracts obtained from this species.

*Pelargonium x fragrans,* a natural hybrid between *P. odoratissimum* and *P. exstipulatum*, is commonly known as nutmeg-scented geranium. Its essential oil does not contain citronellol and geraniol^24^ but is mainly composed of methyl eugenol, limonene, α-pinene, fenchone, and thujene^26^. The essential oil demonstrated antimicrobial and spasmolytic effects^26^. Additionally, the ethyl acetate and *n*-butanol extracts from the leaves showed significant antibacterial and antioxidant activities^27^, while coumarins isolated from the acetone extract demonstrated significant antiparasitic, antioxidant, and antifungal effects^28^.

Previous studies have concentrated on analyzing the chemical composition and biological properties of essential oils derived from the three selected *Pelargonium* species^2,25,26^. However, there is little information about the chemistry or biological activities of the organic extracts obtained from these species.

This study aims to provide comprehensive chemical profiles of the non-volatile constituents of the leaves of the three *Pelargonium* species; *P. graveolens, P. denticulatum,* and *P. fragrans* cultivated in Egypt utilizing Ultra-Performance Liquid Chromatography-Mass Spectrometry (UPLC-MS/MS) and The Global Natural Product Social Molecular Networking (GNPS) coupled to multivariate data analysis.

## Materials and methods

### Plant material

Fresh leaves of *P. graveolens, P. denticulatum,* and *P. fragrans* were collected in April 2023 from the Experimental Station of Medicinal and Aromatic Plants, Faculty of Pharmacy, Cairo University, Egypt. Voucher specimens of *P. denticulatum* (O.S.155)*, P. graveolens* (O.S.156)*,* and* P. fragrans* (O.S.157) were placed at the herbarium of the National Research Centre in Egypt.

### Preparation of the crude extracts

The leaves of the plants were dried in the shade and ground into powder. Twenty grams of the powdered leaves of each plant (*P. graveolens, P. denticulatum,* and *P. fragrans*) were separately extracted with 80% aqueous ethanol (100 mL × 3) by maceration. The extraction process was repeated in triplicate to obtain three independent samples for metabolite profiling. The extracts obtained were separately evaporated under reduced pressure to yield 2.0 ± 0.3, 1.5 ± 0.2, and 2.5 ± 0.2 g of dry extract, respectively.

### Chemicals

All chemicals and extraction solvents used in this study were of analytical grade, and the solvents for analysis were of HPLC grade.

### Ultra-Performance Liquid Chromatography (UPLC)

Samples (10 mg each) of the dried extracts were separately dissolved in 10 mL of aqueous methanol (50% v/v) of HPLC-grade and sonicated for 5 min. The metabolites derived from the extracts were separated using RP High Strength Silica (HSS) T3 C18 column (100 mm × 2.1 mm containing 1.8 μm diameter particles) in a Waters UPLC system (Acquity, Waters Corporation, USA)^29^. The injection volume was 2 μL, and the flow rate was adjusted to 0.3 mL/min. The chromatographic conditions were performed as follows: Mobile phase A consisted of water containing 0.1% formic acid, and mobile phase B consisted of acetonitrile containing 0.1% formic acid. The separation gradient was as follows: 0–1 min at 1% mobile phase B; 1–11 min mobile phase B was increased linearly from 1 to 40%; 11–13 min, mobile phase B was increased linearly from 40 to 70%; 13–15 min, mobile phase B was increased linearly from 70 to 99%; 15–16 min, mobile phase B was maintained at 99%; 16.0–17.0 min, mobile phase B was linearly decreased from 99 to 1%; 17.0–20.0 min, mobile phase B was maintained at 1%. The column temperature was 40 °C.

### Tandem mass spectrometry (MS/MS) acquisition

Mass spectra were acquired using a high-resolution Orbitrap mass analyzer (Q Exactive system, Thermo Fisher Scientific, Waltham, MA, USA)^29^. Data acquisition was performed in data-dependent acquisition (DDA) which was obtained in positive and negative modes, with scanning range from 50 to 1500 *m/z*. Following MS^1^ analysis, the five most intense precursor ions were selected for subsequent dissociation. The ion spray voltage and positive ion voltage were both 3500 V, the negative ion voltage was 3000 V, Ion source Heated Electrospray Ionization (HESI), the sheath gas pressure was 40 psi, the auxiliary heating gas pressure was 15 psi, the ion source heating temperature was 300 °C, the collision energy was obtained with a 20-40-60 V cycle, the MS^1^ resolution was 70,000, and the MS^2^ resolution was 17,500.

### GNPS molecular networking and annotation of metabolites

Molecular networking was established by the GNPS online platform (https://gnps.ucsd.edu)^30^. The raw LC–MS/MS data files of both negative and positive modes (.raw) format were converted to (.mzML) format using the MS Convert software^31^. These files were then imported into WinSCP and uploaded to the GNPS platform, following specific parameters such as a fragment ion tolerance of *m/z* (0.5 Da), a minimum number of common fragment ions (4), and a minimum cosine score (0.65)^30^. Subsequently, a search of the bronze spectral library was conducted, with the top 10 hits per spectrum. The annotation of the metabolites relied on cosine score, shared peaks, and different databases such as METLIN^32^, HMDB^33^, and MassBank^34^. The resulting molecular networking visualization was accomplished through the utilization of Cytoscape 3.6.0 software, where the different colors of the nodes represent the presence of a certain metabolite in each sample^35^. The negative GNPS networking is available at https://gnps.ucsd.edu/ProteoSAFe/status.jsp?task=412607600cec4d55b279cff5f1b1c842. The positive GNPS networking is available at https://gnps.ucsd.edu/ProteoSAFe/status.jsp?task=3897e10860a64266a89d82202879550a.

### Multivariate data analysis

The mzML files were brought into MSDial for import and subsequent processing^36^. The resulting data was converted into a CSV file and directly exported to the Metaboanalyst 5 platform (https://www.metaboanalyst.ca/)^37^. The dataset was then pareto-scaled and log-transformed to standardize variables and minimize redundancy. Subsequently, the treated LC–MS data was subjected to different statistical analysis methods, including hierarchical cluster analysis (HCA), principal component analysis (PCA) as unsupervised methods, and Partial Least Squares Discriminant Analysis (PLS-DA) as a supervised method^37^.

## Results and discussion

### Metabolite annotation based on UPLC-MS/MS data and GNPS molecular networking

**Fig. 1:**
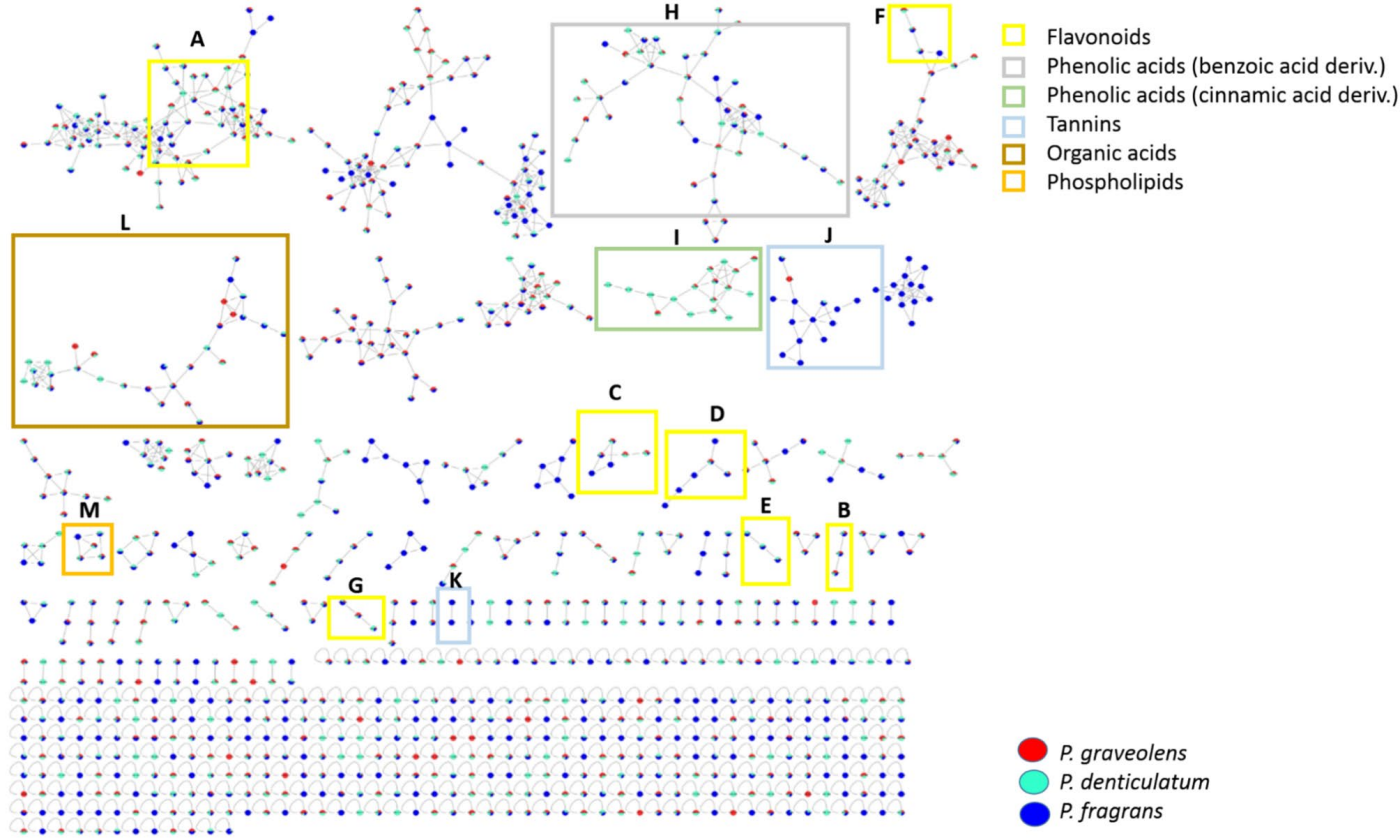
GNPS molecular networking performed using MS/MS data in negative mode. The selected clusters indicate the different classes of the annotated metabolites. A and B, flavonol mono-*O*-glycosides. C, flavonol di- and tri-*O*-glycosides. D, flavone *C*-glycosides. E, flavan-3-ols. F and G, methylated flavonoids. H, phenolic acids (benzoic acid deriv.). I, phenolic acids (cinnamic acid deriv.). J and K, Tannins. L, organic acids. M, phospholipids. Pie charts were used to depict the distribution of ions in *P. graveolens, P. denticulatum,* and* P. fragrans.*

We utilized UPLC-MS/MS to comprehensively analyze the metabolome of the three *Pelargonium* species under investigation; *P. graveolens, P. denticulatum,* and *P. fragrans*. By examining the base peak chromatograms in both ionization modes, distinct chemical profiles for the *Pelargoniums* were observed (Fig. S1). To gain a comprehensive understanding of the chemical space and its distribution, GNPS molecular networks were constructed for both ionization modes. The negative network consisted of 1007 nodes, with 477 nodes grouped into 96 clusters with at least two connected nodes, and 530 features were recognized as single nodes (Fig. 1). In comparison, the positive network contained 2111 nodes, with 1310 nodes connected in 147 clusters, and 801 nodes as self-looped features (Fig. S2). Metabolite annotation was primarily based on retention times, generated chemical formulas, and fragmentation patterns from Xcalibur 2.1 software, and was synchronized with the GNPS. In total, we annotated 154 metabolites, representing various chemical classes such as phenolic acids (benzoic and cinnamic acid derivatives), flavonoids (*O-* and *C-* glycosides), tannins, coumarins, phospholipids, fatty acids, and organic acids. Detailed information on the annotated metabolites, including UPLC-MS/MS data, can be found in Table 1 and Supplemental Data Set 1. The annotation process, as defined by the Metabolomics Standards Initiative (MSI), involved two confidence levels. Confidence level 1 relied on an in-house database of validated standards that were analyzed under the same experimental conditions. Confidence level 2 involved annotating compounds by comparing their spectra to those in existing compound databases or reported Literature^38,39^.

**Table 1:** Tentatively annotated metabolites from the 80% aqueous ethanol extracts of the leaves of *P. graveolens, P. denticulatum,* and *P. fragrans* using UPLC-MS/MS in both negative and positive ionization modes.

Peak no	Tentative identification	R_t_ (min)	*m/z*	Ionization	Molecular formula	MS^2^ ions (Relative intensity)	Error (ppm)	Cosine score
1	Lysine^a^	0.54	147.1126	[M+H]^+^	C_6_H_14_N_2_O_2_	84.0891 (100), 97.0166 (21), 106.0059 (5), 130.0922 (6)	0	0.89
2	Arginine^a^	0.6	175.1191	[M+H]^+^	C_6_H_14_N_4_O_2_	70.0794 (100), 116.0791 (10)	− 0.2	0.93
3	Tartaric acid ^f^	0.61	149.0093	[M–H]^−^	C_4_H_6_O_6_	87.0182 (61), 105.0289 (3), 131.0048 (2)	0.9	0.82
4	Mucic acid^b^	0.62	209.0249	[M–H]^−^	C_6_H_10_O_8_	85.0396 (100), 133.0139 (13), 153.2296 (2), 165.6097 (2), 191.0233 (7)	2.8	0.85
5	Threonic acid^b^	0.64	135.0270	[M–H]^−^	C_4_H_8_O_5_	55.6592 (3), 73.0058 (35), 91.0156 (5), 99.0565 (3)	0.6	0.78
6	Glutamine^a^	0.65	147.0765	[M+H]^+^	C_5_H_10_N_2_O_3_	56.0594 (12), 84.0463 (100), 102.0551 (12), 130.0437 (80)	− 0.1	0.97
7	Xylonic acid^b^	0.65	165.0397	[M−H]^−^	C_5_H_10_O_6_	75.0199 (100), 85.0378 (2), 129.0277 (3), 147.0387 (3)	0.5	0.91
8	Gluconic acid/Galactonic acid^b^	0.66	195.1525	[M–H]^−^	C_6_H_12_O_7_	133.0185 (5), 151.0213 (45), 177.0589 (16)	2.6	0.88
9	Valine^a^	0.66	118.0864	[M+H]^+^	C_5_H_11_NO_2_	55.9482 (20), 59.0063 (46), 69.0032 (9), 72.9472 (52), 100.0257 (100)	− 0.1	0.76
10	Proline^a^	0.71	116.0709	[M+H]^+^	C_5_H_9_NO_2_	70.0673 (100)	0.1	0.83
11	Quinic acid^a^	0.71	191.0555	[M–H]^−^	C_7_H_12_O_6_	129.0274 (3), 147.0561 (4), 173.0252 (100)	1.5	0.96
12	Glucuronic acid/Galacturonic acid^b^	0.72	193.0347	[M–H]^−^	C_6_H_10_O_7_	131.0341 (100), 139.7971 (9), 175.0294 (21)	0.5	0.94
13	Fumaric acid^a^	0.73	115.0026	[M–H]^−^	C_4_H_4_O_4_	53.0893 (2), 71.0181 (2), 97.9374 (21)	0.1	0.86
14	Malic acid^b^	0.73	133.0073	[M–H]^−^	C_4_H_6_O_5_	71.0157 (100), 89.0273 (6), 115.3959 (6)	0	0.95
15	Shikimic Acid^b^	0.73	173.0448	[M–H]^−^	C_7_H_10_O_5_	93.0336 (100), 111.0162 (11)	2.5	0.79
16	Galloyl hexoside^f^	1.18	331.0728	[M–H]^−^	C_13_H_16_O_10_	125.4273 (41), 169.9197 (100)	2.2	0.77
17	Iso/Citric acid^a^	1.31	191.0192	[M–H]^−^	C_6_H_8_O_7_	111.0970 (13), 129.0332 (5), 147.0871 (4), 155.0195 (7), 173.2935 (100)	− 1.7	0.81
18	5-oxo-l-proline^b^	1.45	130.0500	[M+H]^+^	C_5_H_7_NO_3_	84.0649 (100)	0.1	0.92
19	Succinic acid^a^	1.7	117.0184	[M–H]^−^	C_4_H_6_O_4_	55.3956 (2), 73.0639 (10), 99.9137 (100)	0	0.87
20	Adenosine^a^	2.6	268.1041	[M + H]^+^	C_10_H_13_N_5_O_4_	136.1287 (100)	− 0.5	0.98
21	Mucic acid *O*-gallate^c^	2.6	361.0329	[M–H]^−^	C_13_H_14_O_12_	125.0943 (65), 169.0349 (100), 209.9485 (20)	− 1.4	0.75
22	Pyrogallol^c^	2.6	125.0234	[M–H]^−^	C_6_H_6_O_3_	69.0849 (24), 78.9635 (100)	0.6	0.90
23	Trihydroxy benzoic acid (Gallic acid)^e^	2.6	169.0135	[M–H]^−^	C_7_H_6_O_5_	69.0359 (17), 79.0265 (5), 125.0237 (100)	2.2	0.84
24	Guanosine^b^	2.71	282.0848	[M–H]^−^	C_10_H_13_N_5_O_5_	150.0277 (100)	0	0.99
25	Leonuriside A^d^	3.03	331.1037	[M–H]^−^	C_14_H_20_O_9_	139.0453 (5), 169.0182 (30)	1.3	0.89
26	Epi/Gallocatechin-*O*-glucuronide^c^	3.12	481.0996	[M–H]^−^	C_21_H_22_O_13_	125.0291 (12), 179.0277 (2), 219.0676 (4), 261.0286 (2), 305.0657 (100)	1.3	0.95
27	Mucic acid lactone-*O*-gallate^c^	3.17	343.0684	[M–H]^−^	C_13_H_12_O_11_	125.0212 (33), 169.0175 (100), 191.0629 (19)	2.8	0.93
28	Phenylalanine^a^	3.17	166.0861	[M+H]^+^	C_9_H_11_NO_2_	91.0595 (8), 95.0524 (9), 103.0588 (52), 120.0859 (100)	0.6	0.78
29	Epi/Gallocatechin^f^	3.23	305.0720	[M–H]^−^	C_15_H_14_O_7_	125.0261 (100), 179.0374 (18), 205.0422 (22), 219.0714 (17), 245.0783 (50), 289.0721 (6)	1.5	0.81
30	Epi/Gallocatechin-epi/gallocatechin^f^	3.33	609.1269	[M–H]^−^	C_30_H_26_O_14_	305.0745 (70), 423.0782 (100), 441.0146 (49), 473.0148 (4), 541.0451 (7), 591.1266 (13)	− 2.6	0.97
31	Glutaric acid^b^	3.34	131.0340	[M–H]^−^	C_5_H_8_O_4_	69.0323 (3), 87.0455 (100), 113.0239 (6)	0.2	0.92
32	Caffeoyl tartaric acid ^c^	3.38	311.1352	[M–H]^−^	C_13_H_12_O_9_	149.0287 (100), 179.0624 (64)	0.8	0.86
33	Dihydroxy coumarin hexoside^c^	3.53	339.0725	[M–H]^−^	C_15_H_16_O_9_	97.0158 (2), 133.0371 (45), 149.0122 (4), 159.0648 (12), 177.0251 (24)	1.3	0.94
34	Dihydroxy benzoic acid-*O*-hexoside^c^	3.56	315.0763	[M–H]^−^	C_13_H_16_O_9_	153.0074 (80)	1.4	0.77
35	Epi/Gallocatechin-epi/catechin^c^	3.6	593.1312	[M–H]^−^	C_30_H_26_O_13_	177.0295 (12), 289.0657 (13), 303.0582 (6), 407.0932 (16), 467.1085 (25), 425.0973 (15)	− 2.7	0.85
36	Pantothenic acid^a^	3.64	218.1032	[M–H]^−^	C_9_H_17_NO_5_	72.0147 (3), 88.0491 (100), 99.0425 (14), 146.0725 (61)	1.6	0.80
37	Dihydroxy benzoic acid (Protocatechuic acid)^e^	3.72	153.0185	[M−H]^−^	C_7_H_6_O_4_	109.0372 (100)	1.5	0.96
38	Hydroxy benzoic acid^c^	3.88	137.0235	[M−H]^−^	C_7_H_6_O_3_	75.0193 (44), 93.0039 (100)	0.1	0.79
39	Methyl *O*-galloyl-hexoside^c^	3.94	345.1071	[M−H]^−^	C_14_H_18_O_10_	125.0286 (3), 169.0172 (7), 183.0329 (100)	1.4	0.88
40	Galloyl prodelphinidin B1/B2^d^	4.05	761.1370	[M−H]^−^	C_37_H_30_O_18_	305.0791 (57), 42.0712 (100), 441.0789 (47), 591.1233 (39), 609.1375 (66)	2.7	0.91
41	Dihydroxy cinnamic acid^c^	4.13	179.0343	[M−H]^−^	C_9_H_8_O_4_	135.0409 (54), 161.0242 (7)	0.4	0.83
42	Mucic acid lactone-di-*O*-gallate^d^	4.15	495.0878	[M−H]^−^	C_20_H_16_O_15_	125.0242 (6), 169.0124 (100), 191.0468 (12), 343.0719 (43)	− 3.3	0.76
43	Adipic acid^d^	4.19	145.0498	[M−H]^−^	C_6_H_10_O_4_	83.0562 (26), 101.0643 (100), 127.0441 (4)	0.1	0.98
44	Tryptophan^a^	4.2	203.0823	[M−H]^−^	C_11_H_12_N_2_O_2_	116.0586 (100), 130.0739 (4)	− 0.2	0.90
45	Iso/Salicin^b^	4.23	285.0622	[M−H]^−^	C_13_H_18_O_7_	93.0351 (2), 107.0496 (90), 123.0481 (34)	1.3	0.82
46	Hydroxy methoxy benzoic acid-*O*-hexoside^c^	4.27	329.0884	[M−H]^−^	C_14_H_18_O_9_	59.0156 (100), 109.0271 (8), 123.0435 (24), 167.0327 (88)	1.5	0.89
47	*O*-Caffeoyl hexoside^c^	4.29	341.0882	[M−H]^−^	C_15_H_18_O_9_	161.0259 (6), 179.0817 (100)	1.2	0.75
48	Dihydroxy coumarin^c^	4.33	177.0187	[M−H]^−^	C_9_H_6_O_4_	97.0141 (13), 133.0257 (3), 149.0053 (60), 159.1227 (6)	− 0.5	0.95
49	Epi/Catechin hexoside^c^	4.45	451.1253	[M−H]^−^	C_21_H_24_O_11_	204.0764 (5), 277.0634 (15), 289.0721 (100)	1.3	0.97
50	Syringic acid *O*-hexosyl ester^c^	4.51	359.0992	[M−H]^−^	C_15_H_20_O_10_	122.0436 (12), 153.0543 (13), 197.0763 (100)	− 1.6	0.84
51	Hydroxy coumarin (Umbelliferone)^e^	4.63	161.0448	[M−H]^−^	C_9_H_6_O_3_	115.0221 (12), 117.0563 (33), 133.0247 (70), 143.0002 (21)	− 2	0.79
52	Sinapoyl hexoside^c^	4.66	385.0782	[M−H]^−^	C_17_H_22_O_10_	208.0484 (25), 223.0628 (100)	1.8	0.87
53	Di-*O*-galloyl quinic acid^a^	4.69	495.0790	[M−H]^−^	C_21_H_20_O_14_	125.0243 (4), 169.0164 (92), 191.0604 (60), 343.0809 (100)	2.5	0.93
54	Di-*O*-galloyl hexoside^c^	4.73	483.0787	[M−H]^−^	C_20_H_20_O_14_	125.0216 (11), 169.0174 (82), 313.0639 (15)	3.5	0.96
55	*O*-Caffeoyl dihydroxy phenyl lactic acid^c^	4.73	359.0918	[M−H]^−^	C_18_H_16_O_8_	135.0225 (12), 161.0858 (3), 179.0344 (60), 197.1227 (45)	0.9	0.91
56	Darendoside A^d^	4.77	431.1565	[M−H]^−^	C_19_H_28_O_11_	119.0254 (13), 137.1417 (5), 299.1503 (33)	− 1.5	0.76
57	*O*-Caffeoyl hexoside isomer 1^c^	4.86	341.0880	[M−H]^−^	C_15_H_18_O_9_	161.0447 (14), 179.0639 (100)	0.2	0.88
58	*O*-Caffeoyl quinic acid^c^	4.9	353.0682	[M−H]^−^	C_16_H_18_O_9_	179.0287 (75), 191.0576 (44)	3.8	0.83
59	Methyl gallate^c^	4.93	183.0282	[M−H]^−^	C_8_H_8_O_5_	125.0257 (24), 169.0433 (40)	− 2.5	0.94
60	Epi/Catechin^c^	4.97	289.0721	[M−H]^−^	C_15_H_14_O_6_	179.0325 (15), 205.0524 (25), 245.0714 (54)	1.1	0.77
61	Crosatoside B^d^	5	445.1722	[M−H]^−^	C_20_H_30_O_11_	119.0305 (4), 137.0448 (12), 299.0562 (7)	1.3	0.82
62	Coumarin^d^	5.04	147.0440	[M + H]^+^	C_9_H_6_O_2_	103.0446 (5), 119.0585 (11), 129.0135 (6)	− 2.8	0.98
63	*p*-Coumaroyl hexoside^f^	5.04	325.0933	[M−H]^−^	C_15_H_18_O_8_	101.0662 (3), 119.0148 (12), 163.0121 (60)	1.3	0.90
64	Hydroxy cinnamic acid^c^	5.04	163.0393	[M−H]^−^	C_9_H_8_O_3_	119.0257 (100)	0.3	0.85
65	Phlorisobutyrophenone-hexoside^d^	5.07	357.1197	[M−H]^−^	C_16_H_22_O_9_	149.0127 (3), 163.0362 (5), 177.0466 (12), 195.0529 (65)	1.3	0.79
66	*O*-Caffeoyl quinic acid isomer ^c^	5.2	353.0681	[M−H]^−^	C_16_H_18_O_9_	179.0294 (90), 191.0663 (15)	1.8	0.92
67	Pimelic acid^d^	5.21	159.0654	[M−H]^−^	C_7_H_12_O_4_	97.0241 (4), 115.0312 (65), 141.0344 (12)	0.4	0.96
68	Tri-*O*-galloyl hexoside^c^	5.27	635.0902	[M−H]^−^	C_27_H_24_O_18_	125.0239 (3), 169.0166 (33), 313.0591 (6), 483.0807 (7)	2.7	0.89
69	Comososide^d^	5.3	327.1089	[M−H]^−^	C_16_H_24_O_7_	71.0227 (4), 89.0437 (15), 101.0423 (3), 161.0672 (6), 109.0324 (42), 123.0223 (56), 137.1068 (33), 165.1116 (6)	3.9	0.95
70	Feruloyl tartaric acid^c^	5.31	325.0569	[M−H]^−^	C_14_H_14_O_9_	149.0148 (11), 193.0582 (100)	2.5	0.75
71	Dihydroxy methoxy coumarin hexoside (Fraxin)^e^	5.45	369.0831	[M−H]^−^	C_16_H_18_O_10_	127.0223 (3), 145.0393 (4), 163.0411 (16), 177.0433 (33), 179.0568 (4), 189.0525 (34), 207.0704 (44)	1.2	0.81
72	*O*-Caffeoyl hexoside isomer 2^c^	5.46	341.0880	[M−H]^−^	C_15_H_18_O_9_	161.0226 (75), 179.0785 (100)	1.5	0.93
73	Corilagin^d^	5.53	633.0749	[M−H]^−^	C_27_H_22_O_18_	125.0357 (2), 169.0184 (12), 301.0012 (37), 463.0594 (9), 615.0711 (4)	1.4	0.84
74	Dihydroxy methoxy coumarin (Fraxetin)^e^	5.55	207.0293	[M−H]^−^	C_10_H_8_O_5_	127.0222 (3), 145.0482 (12), 163.0072 (56), 177.0552 (45), 179.0202 (5), 189.0109 (6)	− 2.1	0.77
75	Epi/Gallocatechin gallate ([2R,3S]-epigallocatechin-3-gallate)^e^	5.73	457.0783	[M−H]^−^	C_22_H_18_O_11_	125.0284 (24), 169.0116 (100), 305.0796 (9)	− 2.1	0.86
76	*p*-Coumaroyl dihydroxy phenyl lactic acid^c^	5.73	343.0827	[M−H]^−^	C_18_H_16_O_7_	163.0465 (12), 197.0067 (100)	0.8	0.97
77	Tri-*O*-galloyl hexoside isomer^c^	5.74	635.0897	[M−H]^−^	C_27_H_24_O_18_	125.0217 (1), 169.0123 (11), 313.0635 (4), 483.0654 (6)	− 1.7	0.90
78	Isookanin-*O*-hexoside^c^	5.75	449.1098	[M−H]^−^	C_21_H_22_O_11_	107.0532 (2), 125.0297 (45), 151.0074 (6), 243.0769 (6), 259.0673 (64), 287.0696 (100)	1.4	0.88
79	Ellagic acid hexoside^b^	5.78	463.0526	[M−H]^−^	C_20_H_16_O_13_	169.0151 (2), 301.0054 (100)	3.3	0.79
80	Heterophylliin A^d^	6.01	785.0862	[M−H]^−^	C_34_H_26_O_22_	125.0322 (6), 169.0196 (12), 301.0087 (50), 463.0568 (7), 651.0663 (8)	2.5	0.92
81	*p*-Coumaroyl quinic acid^a^	6.02	337.0571	[M−H]^−^	C_16_H_18_O_8_	163.0456 (100), 191.0326 (19)	2	0.76
82	Myricetin-*O*-hexosyl-deoxyhexoside^f^	6.1	625.1417	[M−H]^−^	C_27_H_30_O_17_	179.0039 (2), 316.0294 (35), 317.0363 (8)	2.7	0.95
83	Quercetin-*O*-hexosyl-deoxyhexosyl*-*deoxyhexoside^b^	6.15	755.2056	[M−H]^−^	C_33_H_40_O_20_	121.0179 (2), 151.0427 (68), 179.0059 (100), 300.0321 (3), 301.1253 (4)	2.6	0.83
84	Mucic acid lactone methyl ester-*O-*gallate^d^	6.17	357.0620	[M−H]^−^	C_14_H_14_O_11_	125.0181 (4), 169.0011 (100), 191.0232 (10), 343.0691 (13)	-1.3	0.94
85	Quercetin-*O*-hexosyl-hexoside^b^	6.21	625.1420	[M−H]^−^	C_27_H_30_O_17_	121.0361 (1), 151.0242 (33), 179.0151 (100), 300.0194 (2), 301.0276 (1)	2.5	0.85
86	Quercetin-*O*-hexoside *O*-hexoside^b^	6.31	625.1422	[M−H]^−^	C_27_H_30_O_17_	151.0048 (44), 179.0232 (65), 300.0206 (1), 301.0329 (2), 463.0921 (27)	− 2.5	0.98
87	Myricetin-*O*-hexoside^f^	6.35	479.0836	[M−H]^−^	C_21_H_20_O_13_	61.9927 (5), 179.0083 (4), 316.0241 (100), 317.0353 (16)	0.7	0.88
88	Di-*O*-methyl mucic acid lactone-*O*-gallate^d^	6.37	371.0987	[M−H]^−^	C_15_H_16_O_11_	125.0257 (3), 169.0913 (100), 191.0657 (2), 357.0461 (2)	− 2.1	0.81
89	Luteolin-*C*-hexoside (Iso Orientin/Orientin)^e^	6.37	447.0948	[M−H]^−^	C_21_H_20_O_11_	133.0334 (2), 175.0277 (12), 267.0502 (10), 284.0372 (3), 285.0431 (6), 327.0582 (100), 357.0692 (32)	3.5	0.75
90	Quercetin *O*-hexoside *O*-pentoside^f^	6.43	595.1334	[M−H]^−^	C_26_H_28_O_16_	151.0235 (2), 179.0005 (3), 300.0344 (60), 301.0469 (10), 433.0678 (3), 463.0957 (4)	2.6	0.89
91	Ethyl gallate^b^	6.48	197.0451	[M−H]^−^	C_9_H_10_O_5_	125.0339 (9), 169.0113 (100)	0.5	0.93
92	Hydroxy methoxy benzoic acid ^c^	6.54	167.0342	[M−H]^−^	C_8_H_8_O_4_	108.0067 (12), 123.0147 (100), 152.0461 (4)	0.3	0.82
93	Taxifolin-*O*-hexosyl-deoxyhexoside^c^	6.54	611.1396	[M−H]^−^	C_27_H_32_O_16_	151.0094 (2), 179.0062 (3), 303.0424 (40)	2.5	0.90
94	Kaempferol-*O*-hexosyl-deoxyhexosyl-deoxyhexoside^b^	6.55	739.2119	[M−H]^−^	C_33_H_40_O_19_	229.4457 (5), 243.0139 (2), 257.0261 (2), 284.0387 (100), 285.0409 (33)	2.6	0.77
95	Tetra-*O*-galloyl hexoside^b^	6.57	787.1014	[M−H]^−^	C_34_H_28_O_22_	169.0116 (16), 465.0782 (32), 483.0668 (12), 617.0772 (100), 635.0706 (75)	2	0.96
96	Isorhamnetin-*O*-hexosyl-deoxyhexosyl-deoxyhexoside^b^	6.64	769.2208	[M−H]^−^	C_34_H_42_O_20_	67.9528 (4), 68.4829 (5), 68.6332 (4), 86.0219 (5), 91.9262 (5), 138.4329 (5), 177.4088 (6), 305.2992 (6), 314.0429 (100), 315.0578 (19)	1.9	0.84
97	Suberic acid^d^	6.64	173.0812	[M−H]^−^	C_8_H_14_O_4_	111.0106 (6), 129.0009 (100), 155.0261 (22)	0.4	0.86
98	Myricetin-*O*-pentoside^f^	6.73	449.0732	[M−H]^−^	C_20_H_18_O_12_	151.0022 (1), 179.0052 (3), 242.0245 (1), 316.0414 (100), 317.0112 (7)	2.8	0.81
99	Quercetin-*O*-hexosyl-deoxyhexoside^f^	6.81	609.1489	[M−H]^−^	C_27_H_30_O_16_	151.0051 (12), 179.0008 (5), 300.0356 (90), 301.0409 (40)	1.9	0.92
100	Myricetin-*O*-deoxyhexoside^f^	6.87	463.0887	[M−H]^−^	C_21_H_20_O_12_	151.0013 (1), 179.0201 (2), 316.0148 (100), 317.0321 (12)	1.6	0.79
101	Ellagic acid^b^	6.89	300.9991	[M−H]^−^	C_14_H_6_O_8_	185.0272 (5), 229.0125 (7), 257.0175 (4)	1.1	0.95
102	Apigenin-*C*-hexoside^b^	6.91	431.0995	[M−H]^−^	C_21_H_20_O_10_	269.0574 (6), 311.0601 (100), 341.0739 (29)	3.7	0.97
103	Quercetin-*O*-hexoside^f^	6.95	463.0888	[M−H]^−^	C_21_H_20_O_12_	121.0178 (5), 151.0037 (4), 179.0082 (1), 300.0441 (65), 301.0285 (90)	− 1.5	0.88
104	Kaempferol-*O*-hexoside *O*-pentoside^b^	6.98	579.1364	[M−H]^−^	C_26_H_28_O_15_	151.0073 (1), 211.0146 (1), 243.0459 (2), 284.0316 (80), 285.0437 (18), 447.0967 (2)	0.9	0.75
105	Quercetin-*O*-hexoside isomer^f^	7.04	463.0888	[M−H]^−^	C_21_H_20_O_12_	121.0281 (4), 151.0002 (4), 179.0712 (2), 300.0387 (70), 301.0004 (100)	1.2	0.83
106	Kaempferol galloyl hexoside d	7.09	599.1055	[M−H]^−^	C_28_H_24_O_15_	125.0298 (2), 169.0169 (25), 229.0547 (2), 243.0458 (2), 257.0377 (1), 285.0684 (23), 437.0134 (12)	− 1.5	0.91
107	Luteolin-*O*-hexoside^b^	7.1	447.0938	[M−H]^−^	C_21_H_20_O_11_	133.2287 (1), 175.0481 (3), 199.0275 (1), 217.0365 (2), 241.0139 (5), 284.0395 (25), 285.0498 (100)	1.4	0.94
108	Kaempferol-*O*-hexosyl-deoxyhexoside^f^	7.17	593.1525	[M−H]^−^	C_27_H_30_O_15_	151.0001 (1), 229.0374 (5), 243.0159 (5), 257.0129 (12), 285.1096 (100)	2.9	0.78
109	Kaempferol-*O*-hexosyl-deoxyhexoside isomer^b^	7.37	593.1520	[M−H]^−^	C_27_H_30_O_15_	243.0589 (1), 257.0429 (4), 284.0194 (69), 285.0955 (100)	− 2.7	0.97
110	Quercetin-*O*-pentoside^f^	7.4	433.0780	[M−H]^−^	C_20_H_18_O_11_	121.0148 (2), 151.0245 (2), 179.06 (3), 300.0377 (100), 301.0481 (60)	1.4	0.90
111	Isorhamnetin-*O*-hexosyl-deoxyhexoside^b^	7.52	623.1627	[M−H]^−^	C_28_H_32_O_16_	300.0958 (11), 314.1089 (45), 315.1178 (100)	3	0.86
112	Kaempferol-*O*-hexoside^f^	7.63	447.0936	[M−H]^−^	C_21_H_20_O_11_	151.0682 (4), 199.0257 (3), 243.0172 (6), 284.0563 (85), 285.0684 (100)	1.4	0.76
113	Naringenin-*O*-hexoside^b^	7.78	433.1145	[M−H]^−^	C_21_H_22_O_10_	107.1078 (1), 119.0953 (6), 151.0002 (45), 271.0682 (100)	0.2	0.85
114	Kaempferol-*O*-pentoside^f^	7.87	417.0834	[M−H]^−^	C_20_H_18_O_10_	151.0157 (2), 199.0746 (3), 211.0177 (2), 243.0458 (2), 284.0194 (45), 285.0684 (100)	1.6	0.82
115	Azelaic acid^d^	8	187.0970	[M−H]^−^	C_9_H_16_O_4_	97.0685 (17), 125.1083 (100)	0.5	0.98
116	Isorhamnetin-*O*-hexoside^b^	8.07	477.1044	[M−H]^−^	C_22_H_22_O_12_	300.0589 (12), 314.0953 (65), 315.1288 (100)	0.1	0.79
117	Myricetin^f^	8.11	317.0305	[M−H]^−^	C_15_H_10_O_8_	151.0001 (60), 179.02 (50), 227.0291 (21), 317.0305 (100)	1.3	0.88
118	Isookanin^c^	8.15	287.0565	[M−H]^−^	C_15_H_12_O_6_	107.0149 (6), 125.0288 (100), 151.0001 (13), 243.0778 (11), 259.0657 (59)	0.8	0.93
119	Methoxy hydroxy cinnamic acid ^c^	8.28	193.0501	[M−H]^−^	C_10_H_10_O_4_	133.0179 (23), 149.0243 (75), 161.0003 (100), 178.0286 (13)	0.5	0.96
120	Kaempferol-*O*-deoxyhexoside^f^	8.41	431.0989	[M−H]^−^	C_21_H_20_O_10_	151.0178 (6), 199.0481 (3), 211.0487 (2), 243.0982 (4), 284.0395 (77), 285.0498 (100)	2.1	0.91
121	Hydroxy methoxy coumarin^c^	8.63	191.0345	[M−H]^−^	C_10_H_8_O_4_	129.0645 (5), 145.0481 (4), 147.0281 (12), 161.0002 (100), 163.0148 (45), 173.0187 (12)	2.8	0.75
122	Methoxy coumarin^c^	8.76	175.0606	[M−H]^−^	C_10_H_8_O_3_	131.0264 (100), 145.0589 (23)	− 1.6	0.84
123	Sebacic acid^d^	9.33	201.1128	[M−H]^−^	C_10_H_18_O_4_	139.1281 (2), 157.1486 (20), 183.0298 (50)	0.8	0.77
124	Hydroxy decanoic acid^b^	9.35	187.1334	[M−H]^−^	C_10_H_20_O_3_	125.1096 (100), 143.1179 (5)	2.2	0.89
125	Luteolin^b^	9.41	285.0407	[M−H]^−^	C_15_H_10_O_6_	133.0187 (2), 151.0289 (3), 175.0487 (33), 199.0275 (16), 217.0365 (20), 241.0249 (55), 267.0148 (2)	1.2	0.82
126	Quercetin^f^	9.43	301.0357	[M−H]^−^	C_15_H_10_O_7_	107.0179 (28), 121.0375 (31), 151.0002 (100), 179.0721 (37), 273.0487 (7)	1.2	0.95
127	Quercetin methyl ether (Isorhamnetin)^f^	9.92	315.0273	[M−H]^−^	C_16_H_12_O_7_	135.0157 (1), 243.0375 (8), 254.0284 (2), 255.0395 (23), 271.0148 (48), 272.0375 (2), 300.0387 (100)	1.3	0.97
128	Naringenin^b^	10.37	271.0616	[M−H]^−^	C_15_H_12_O_5_	93.0375 (17), 107.0187 (3), 119.0543 (10), 151.0002 (80)	1.3	0.79
129	Apigenin^b^	10.55	269.0571	[M−H]^−^	C_15_H_10_O_5_	151.0178 (6), 159.0487 (2), 184.0375 (6), 201.0157 (45), 225.0281 (60), 241.0589 (2)	− 2.7	0.90
130	Kaempferol^f^	10.67	285.0408	[M−H]^−^	C_15_H_10_O_6_	107.0157 (3), 151.0001 (2), 185.0645 (2), 199.0134 (1), 211.0487 (3), 229.0275 (3), 243.0375 (2), 257.0589 (11), 285.0408 (100)	1.3	0.93
131	Dihydroxy-oxo-octadecenoic acid^b^	10.8	327.2181	[M−H]^−^	C_18_H_32_O_5_	171.1087 (18), 183.1486 (4), 211.1379 (36), 291.2086 (5), 309.2178 (2)	4.1	0.96
132	Kaempferol methyl ether^c^	11.17	299.0783	[M−H]^−^	C_16_H_12_O_6_	229.0487 (51), 243.0375 (50), 257.0487 (5), 284.0395 (46), 285.0498 (100)	2.7	0.76
133	Trihydroxy octadecenoic acid^b^	11.46	329.2338	[M−H]^−^	C_18_H_34_O_5_	171.1087 (12), 211.1379 (43), 229.1486 (24), 293.2178 (2), 311.2281 (2)	3.8	0.88
134	Trihydroxy octadecenoic acid isomer^b^	12.35	329.2336	[M−H]^−^	C_18_H_34_O_5_	171.2086 (10), 211.1379 (42), 229.1486 (20), 311.2487 (3)	3.9	0.81
135	Luteolin methyl ether^c^	12.57	299.0635	[M−H]^−^	C_16_H_12_O_6_	151.0157 (2), 175.0134 (12), 199.0134 (15), 241.0249 (33), 284.0264 (45), 285.0498 (100)	2.8	0.75
136	Apigenin methyl ether^c^	12.73	283.0613	[M−H]^−^	C_16_H_12_O_5_	159.0157 (2), 201.0645 (13), 225.0157 (10), 241.0249 (1), 269.0589 (100)	2.7	0.94
137	Luteolin dimethyl ether^c^	12.75	313.0729	[M−H]^−^	C_17_H_14_O_6_	175.0134 (6), 199.0004 (5), 217.0157 (10), 241.0645 (16), 284.0264 (1), 285.0684 (65), 299.0487 (100)	2	0.85
138	Trihydroxy octadecanoic acid^b^	12.8	331.2493	[M−H]^−^	C_18_H_36_O_5_	277.2291 (3), 295.2395 (3), 313.2487 (10)	4.4	0.98
139	Methoxy luteolin dimethyl ether ^c^	12.83	343.0496	[M−H]^−^	C_18_H_16_O_7_	199.0589 (6), 241.0487 (4), 267.0589 (11), 270.1096 (5), 285.2096 (40), 313.0589 (23), 328.2291 (60)	1.4	0.82
140	Hydroxy dimethoxy coumarin (Umckalin)^e^	12.92	221.1544	[M−H]^−^	C_11_H_10_O_5_	161.0589 (3), 177.1281 (6), 191.0002 (40)	1.6	0.79
141	Lysophosphatidylinositol PI(16:0)^b^	12.93	571.2893	[M−H]^−^	C_25_H_49_O_12_P	241.0157 (14), 255.2395 (30), 315.0589 (10), 391.2395 (5)	3.5	0.77
142	Dihydroxy octadecenoic acid^b^	12.94	313.2387	[M−H]^−^	C_18_H_34_O_4_	183.1096 (30), 277.2291 (11), 295.2395 (10)	4.9	0.90
143	Dihydroxy octadecenoic acid isomer^b^	13.51	313.2389	[M−H]^−^	C_18_H_34_O_4_	183.1486 (52), 277.2291 (5), 295.2395 (13)	4.5	0.88
144	Lysophosphatidylcholine LPC(18:3)^a^	13.55	518.3244	[M + H]^+^	C_26_H_48_NO_7_P	184.0746 (100), 258.1179 (2), 500.3176 (11)	0.4	0.89
145	Lysophosphatidic acid PA(22:6)^a^	13.73	481.2578	[M−H]^−^	C_25_H_39_O_7_P	153.0004 (36), 227.0375 (6), 245.0487 (60), 253.2291 (32), 327.9987 (2)	− 2.2	0.76
146	Dihydroxy octadecanoic acid^b^	13.87	315.2543	[M−H]^−^	C_18_H_36_O_4_	253.2291 (3), 279.2395 (3), 297.2487 (12)	3.9	0.92
147	Hydroxy octadecatrienoic acid^b^	13.87	293.2125	[M−H]^−^	C_18_H_30_O_3_	249.1846 (3), 265.2291 (4), 275.3589 (56)	4.5	0.91
148	Lysophosphatidylethanolamine PE(18:2)^a^	13.89	476.2789	[M−H]^−^	C_23_H_44_NO_7_P	122.9984 (2), 196.0487 (10), 279.2395 (100)	3	0.95
149	Lysophosphatidylcholine LPC(18:2)^a^	13.92	520.3497	[M + H]^+^	C_26_H_50_NO_7_P	184.0645 (100), 258.1379 (5), 502.2395 (16)	− 0.2	0.83
150	Lysophosphatidylethanolamine PE(16:0)^a^	14.15	452.2788	[M−H]^−^	C_21_H_44_NO_7_P	196.0487 (10), 255.2395 (100)	3.2	0.96
151	Lysophosphatidylcholine LPC(16:0)^a^	14.19	496.3499	[M+H]^+^	C_24_H_50_NO_7_P	184.0589 (100), 258.1096 (9), 500.1954 (6)	− 0.3	0.94
152	Hydroxy octadecadienoic acid^b^	14.22	295.2281	[M–H]^−^	C_18_H_32_O_3_	195.1486 (16), 251.1096 (2), 277.2291 (51)	4.1	0.78
153	oxo-Octadecadienoic acid^b^	14.52	293.2125	[M−H]^−^	C_18_H_30_O_3_	185.1281 (13), 221.1543 (3), 249.1846 (3)	4.7	0.97
154	Lysophosphatidylglycerol PG(16:0)^a^	14.69	483.2731	[M−H]^−^	C_22_H_45_O_9_P	153.0178 (18), 227.0375 (5), 255.2395 (23), 391.2281 (45)	3.2	0.81

^a^The metabolite annotation indicates confidence level 1 by using an in-house database of authentic standards that were analyzed under the same experimental conditions.

^b–d^The metabolite annotation indicates confidence level 2 by using specific compound databases such as GNPS, MassBank, or METLIN, respectively.

^e,f^The metabolite annotation indicates confidence level 2 by matching the annotated metabolites with literature data (previously isolated or detected from the genus, respectively).

### Flavonoids

Flavonoids, the main class of compounds annotated in *P. graveolens,* are mostly flavonols. These flavonols are believed to be accountable for the antioxidant and anti-inflammatory effects reported for the plant^19–21^. As well, *P. denticulatum* is rich in flavonoids. The aglycones (represented as individual nodes in GNPS networking) were differentiated depending on their molecular ion, predicted molecular formula, and specific RDA fragmentation^40^. For instance, peaks 117, 126, and 130 in the negative mode, were tentatively identified as myricetin, quercetin, and kaempferol, respectively. Kaempferol (*m/z* 285.0408 [M–H]^−^, C_15_H_9_O_6_^−^) is characterized with RDA fragments at *m/z* 257.0589 ([M–H]^−^–CO^−^), 243.0375 ([M–H]^−^–C_2_H_2_O^−^), and 229.0275 ([M–H]^−^–2CO^−^), when compared to literature^40^. In addition, quercetin (*m/z* 301.0357 [M–H]^−^, C_15_H_9_O_7_^−^) has two interesting base peaks at *m/z* 179.0721 and 121.0375 attempts to bonds 1 and 2 concerned with ^1,2^A^−^ and ^1,2^B^−^ fragments, respectively, followed by the appearance of *m/z* 151.0002 [^1,2^A^−^–CO^−^] (Fig. S3)^40^.

**Fig. 2:**
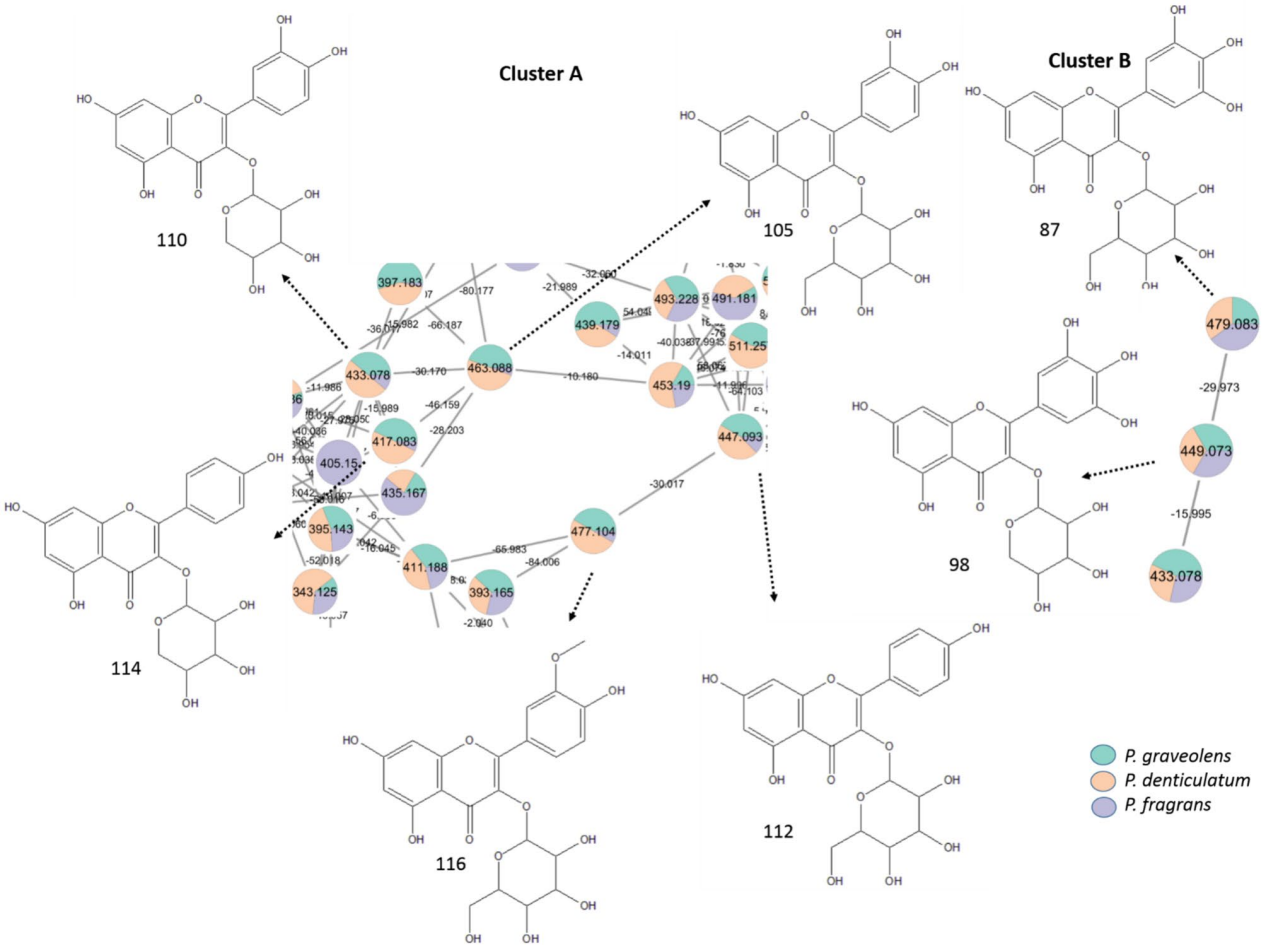
Annotated flavonol mono-*O-*glycosides in the negative mode (position of substitutions may vary) and their distribution in the GNPS molecular network from *P. graveolens, P. denticulatum,* and* P. fragrans.*

**Fig. 3:**
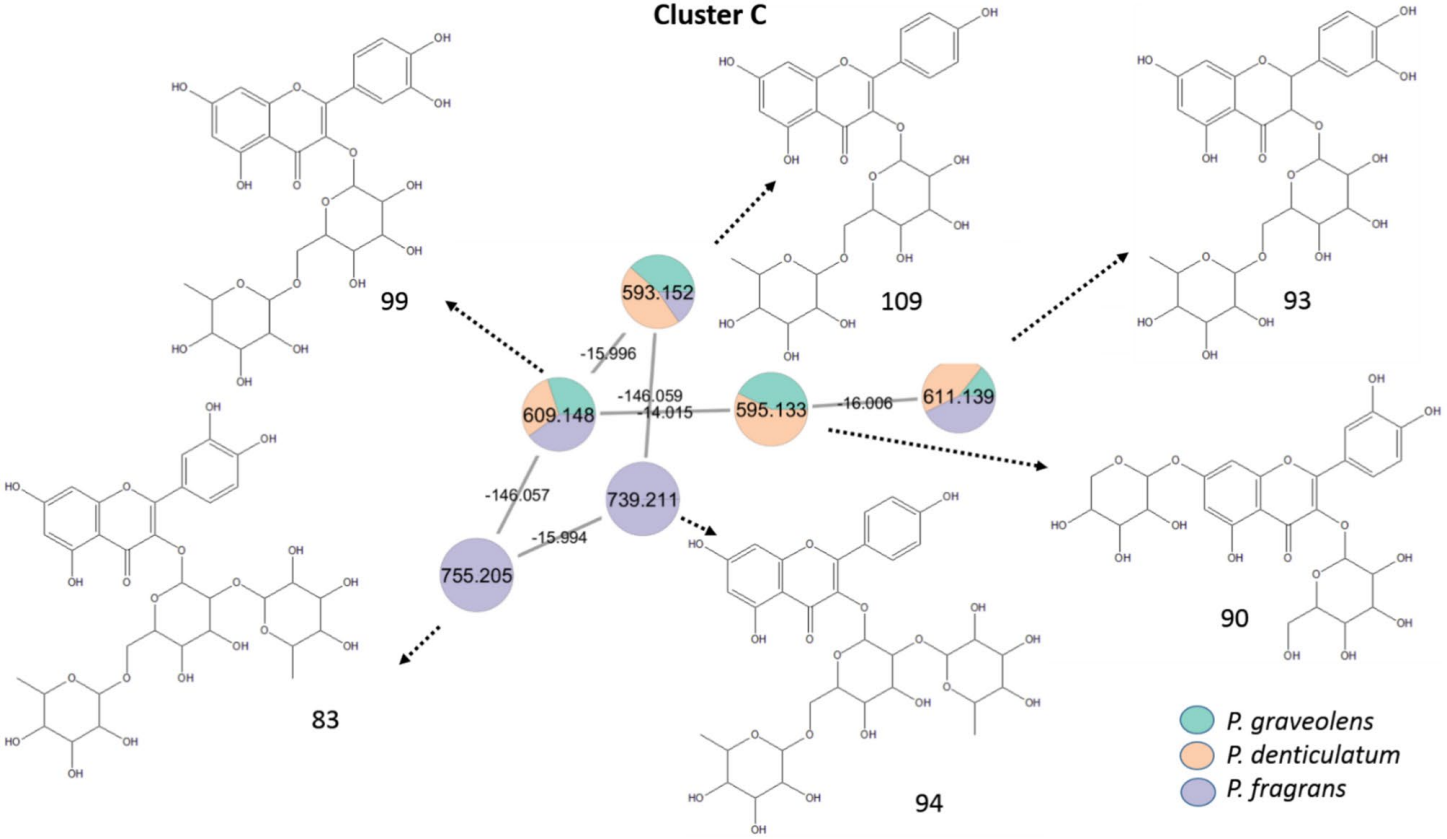
Annotated flavonol di- and tri-*O-*glycosides in the negative mode (position of substitutions may vary) and their distribution in the GNPS molecular network from *P. graveolens, P. denticulatum,* and* P. fragrans.*

Additionally in the negative mode, flavonol mono-*O-*glycosides are observed in clusters A and B (Fig. 2), while flavonol di- and tri-*O-*glycosides are detected in cluster C (Fig. 3). Notably, flavonol tri-*O-*glycosides such as quercetin *O-*hexosyl-deoxyhexosyl*-*deoxyhexoside (83) and kaempferol-*O-*hexosyl-deoxyhexosyl-deoxyhexoside (94) are more predominant in *P. fragrans* than the other two species. In general, sugars in the glycosides were tentatively identified from the values of lost moiety and respective formulas; neutral loss of 132 amu, C_5_H_8_O_4_^−^ for pentosides, 162 amu, C_6_H_10_O_5_^−^ for hexosides, and 146 amu, C_6_H_10_O_4_^−^ for deoxyhexosides^41^.

Peaks 100 and 103 were annotated in the negative mode as myricetin-*O-*deoxyhexoside (*m/z* 463.0887, C_21_H_19_O_12_^−^) and quercetin-*O-*hexoside (*m/z* 463.0888, C_21_H_19_O_12_^−^), respectively. Although having the same [M–H]^−^ and elemental composition, they were tentatively identified based on their pattern of fragmentation, where their respective aglycones, myricetin (*m/z* 317.0321, C_15_H_9_O_8_^−^) and quercetin (*m/z* 301.0285, C_15_H_9_O_7_^−^), appeared after the neutral loss of 146 amu for deoxyhexoside (C_6_H_10_O_4_^−^) and 162 amu for hexoside (C_6_H_10_O_5_^−^), respectively.

On the other side, peak number 90 was tentatively identified as quercetin-*O*-hexoside *O*-pentoside based on the appearance of 2 product ions at *m/z* 463.0957 (for the elimination of a pentosyl moiety; 132 amu) and 433.0678 (for the elimination of a hexosyl moiety; 162 amu) followed by a product ion at *m/z* 301.0469 for the aglycone quercetin. This finding suggested that the 2 sugar moieties are linked to the aglycone at different positions^41^.

**Fig. 4:**
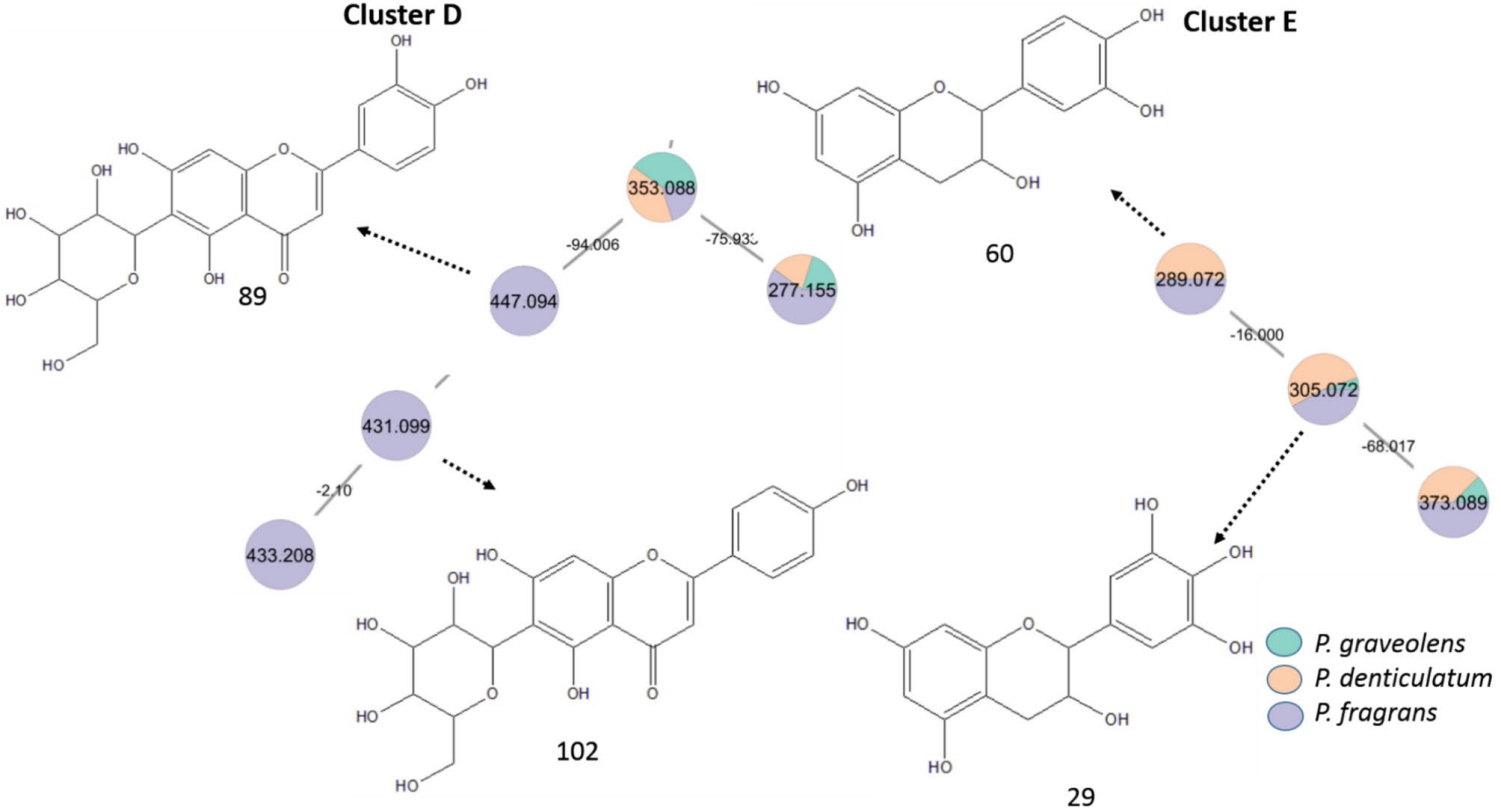
Annotated flavone *C*-glycosides (cluster D) and flavan-3-ols (cluster E) in the negative mode (position of substitutions may vary) with their distribution in the GNPS molecular network from *P. graveolens, P. denticulatum,* and* P. fragrans.*

Flavone *C*-glycosides were more predominantly found in the leaves of *P. fragrans* than in other species (Fig. 4, cluster D). The annotated *C*-glycosides were characterized by the neutral loss of 90 and 120 amu. Peak 102 was tentatively identified as apigenin-*C*-hexoside (*m/z* 431.0995 [M–H]^−^, C_21_H_19_O_10_^−^). On the other side, peak 89 was tentatively identified as luteolin-*C*-hexoside (*m/z* 447.0948 [M–H]^−^, C_21_H_19_O_11_^−^)^42^.

As well, flavan-3-ols such as epi/catechin (60, *m/z* 289.0721 [M–H]^−^, C_15_H_13_O_6_^−^), and epi/gallocatechin (29, *m/z* 305.0720 [M–H]^−^, C_15_H_13_O_7_^−^), were more predominant in *P. denticulatum* and *P. fragrans* (Fig. 4, cluster E), with an edge mass difference of 16 amu and specific RDA fragments at *m/z* 289.0721, 245.0783, and 205.0422^43^*.*

Pointing to the methylated flavonoids (Fig. S4, clusters F and G), they were tentatively identified depending on the neutral loss of 14 amu, CH_2_^−^^44,45^.

Peaks at 128, 118, and 78 were annotated as flavanones; naringenin (*m/z* 271.0616 [M–H]^−^, C_15_H_11_O_5_^−^), isookanin (*m/z* 287.0565 [M–H]^−^, C_15_H_11_O_6_^−^), and isookanin-*O*-hexoside (*m/z* 449.1098 [M–H]^−^, C_21_H_21_O_11_^−^), respectively.

It is noteworthy that many of these flavonoids are being annotated and detected for the first time in the three species. As well, these findings align with the previous study by Williams et al.^46^, which examined 58 species of *Pelargonium*. The study established that flavonols, specifically myricetin, quercetin, and kaempferol, are the primary flavonoid compounds in the leaves of *Pelargonium* species. The species investigated in this study included *P. crispum 'Whiteknights', P. cucullatum, P. graveolens, P. quercifolium,* and *P. tomentosum.* Additionally, *P. crispum 'Whiteknights', P. tomentosum,* and *P. quercifolium* contain isorhamnetin^46^. In addition, (2R,3R)-(+)-dihydroquercetin-3-β-glucopyranoside, isoorientin, and orientin were isolated before from *P. sidoides*^47^.

### Phenolic acids

**Fig. 5:**
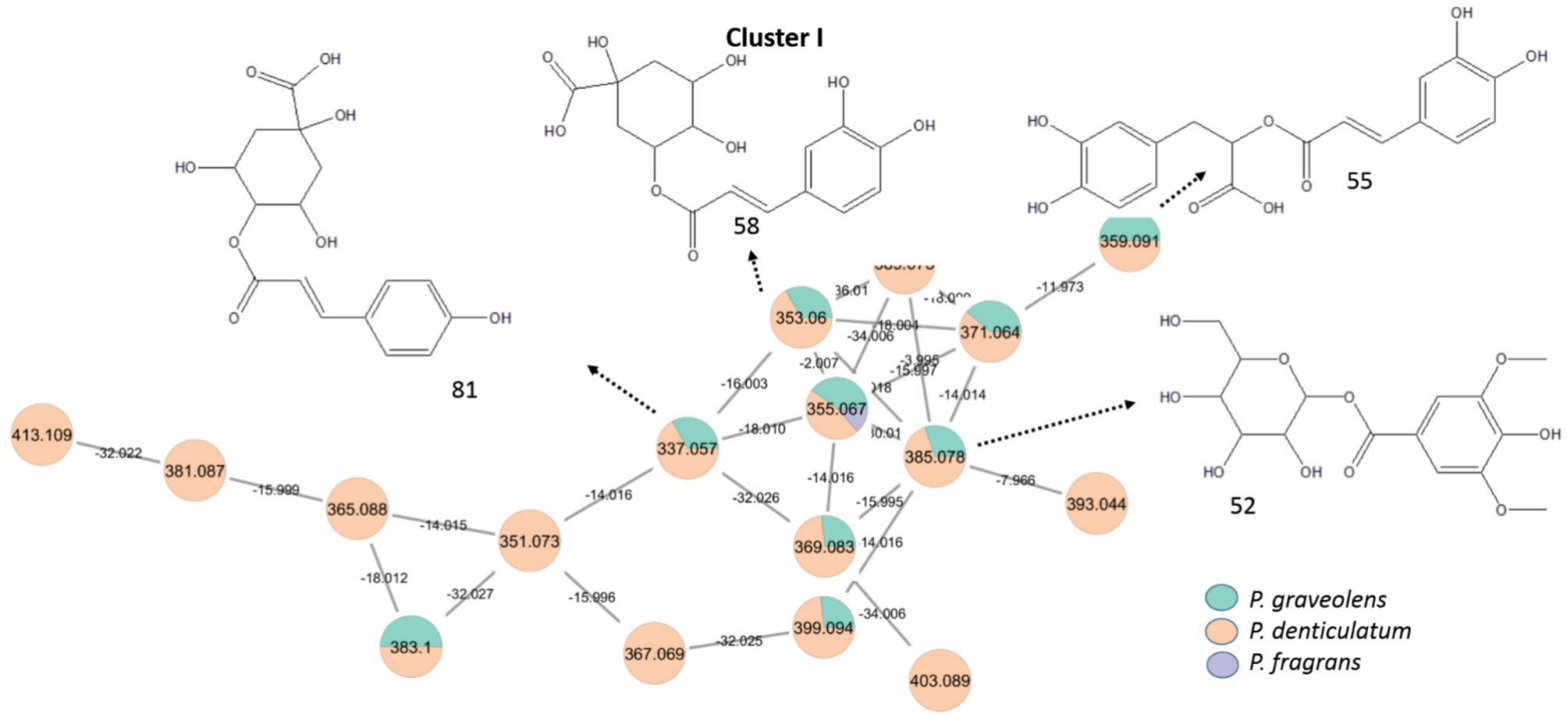
Annotated phenolic acids (cinnamic acid deriv.) in the negative mode (position of substitutions may vary) and their distribution in the GNPS molecular network from *P. graveolens, P. denticulatum,* and* P. fragrans.*

Phenolic acids are secondary metabolites found widely in plants and play an important role in protection against various ailments^48^. This is the first study to examine the profile of phenolic acids in *P. graveolens, P. denticulatum,* and *P. fragrans.* Phenolic acids of the benzoic acid derivatives were predominant in the three species with a unique neutral loss of COO^-^ (44 amu) (Fig. S5, cluster H). On the other hand, phenolic acids of the cinnamic acid derivatives (Fig. 5, cluster I), were tentatively identified in the negative mode to be more predominant in *P. graveolens* and *P. denticulatum* than in *P. fragrans*. Peaks at 58, 66, 70, and 81 were tentatively identified as cinnamic acid derivatives conjugated with organic acids; *O*-caffeoyl quinic acid, *O*-caffeoyl quinic acid isomer, feruloyl tartaric acid, and *p*-coumaroyl quinic acid, respectively, where their MS^2^ data were characterized by the neutral loss of the phenolic acid moiety and the appearance of the ion fragment of the organic acid^49^. For example, peak 58 was tentatively identified as *O*-caffeoyl quinic acid (*m/z* 353.0682 [M-H]^−^, C_16_H_17_O_9_^−^), where its MS^2^ data showed daughter ion fragments at *m/z* 191.0576 of quinic acid (loss of caffeoyl moiety, 162 amu, C_9_H_6_O_3_^-^) and 179.0287 of caffeic acid ion (C_9_H_7_O_4_^−^).

In addition to the cinnamic acid esters, the study also detected the presence of their glycosides; sinapoyl hexoside (52, *m/z* 385.0782 [M–H]^−^, C_17_H_21_O_10_^−^), *O*-caffeoyl hexoside with its two isomers (47, 57 and 72; *m/z* 341.0882 [M–H]^−^; C_15_H_17_O_9_^−^), and *p*-coumaroyl hexoside (63, *m/z* 325.0933 [M–H]^−^, C_15_H_17_O_8_^−^) with a characteristic fragmentation ion at *m/z* 223.0628, 179.0817, and 163.0121, respectively, due to the loss of a hexosyl unit (162 amu, C_6_H_10_O_5_^−^)^50^.

### Tannins

Tannins are a group of polyphenolic compounds that exhibit a wide range of chemical structures and exhibit various therapeutic effects^51^. They can be categorized into different types; hydrolyzable tannins can be broken down through hydrolysis using acids or enzymes into gallic acid or ellagic acid, while condensed tannins are polymers composed of flavan-3-ols units (catechins)^52^. It is also noted that the genus produces both hydrolyzable and non-hydrolyzable tannins in significant quantities^53^. For instance, (2R,3S)-epigallocatechin-3-gallate, gallic acid, and protocatechuic acid were isolated before from *P. sidoides*^47^.

**Fig. 6:**
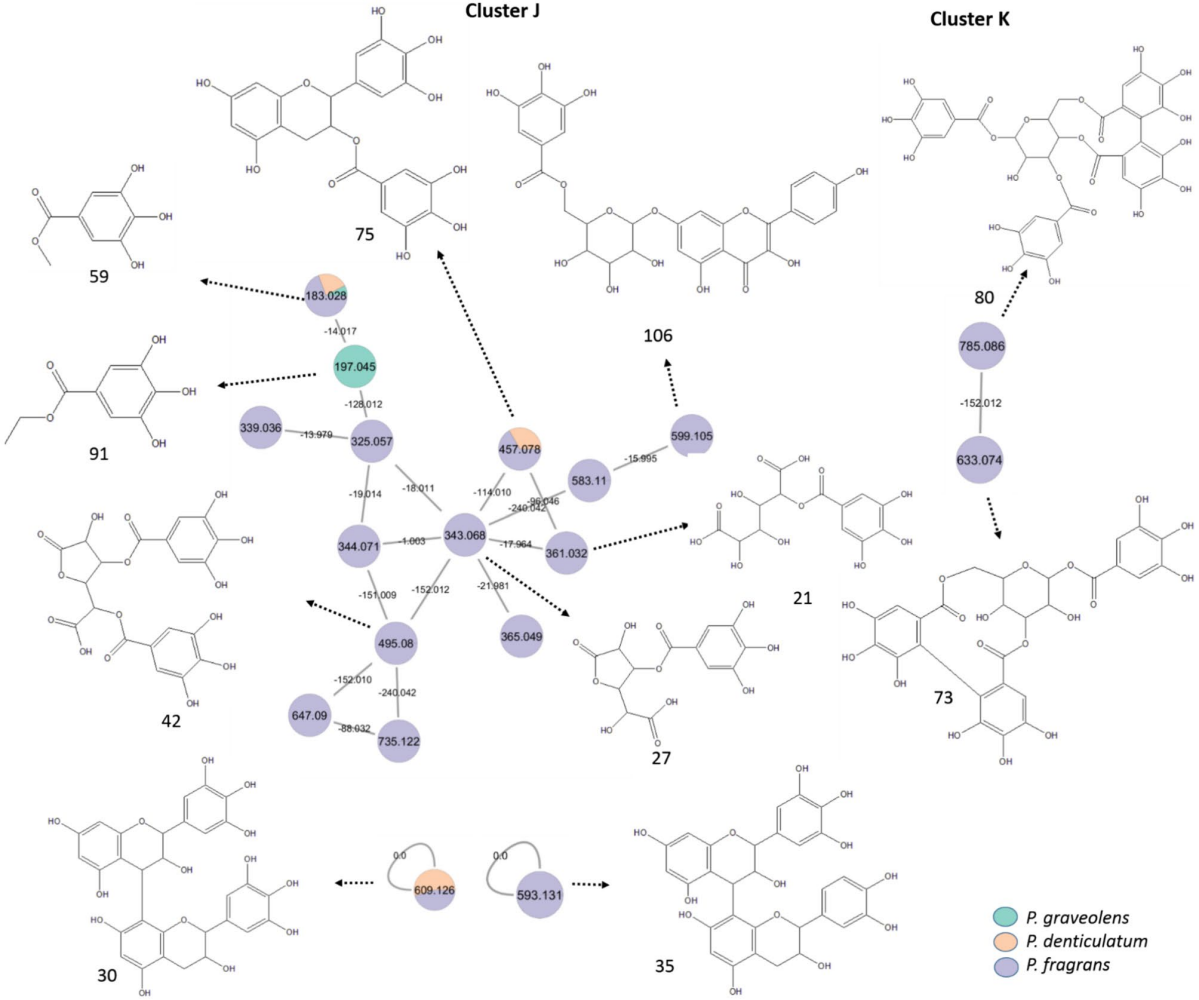
Annotated tannins in the negative mode (position of substitutions may vary) and their distribution in the GNPS molecular network from *P. graveolens, P. denticulatum,* and* P. fragrans.*

Both types of tannins were detected predominantly in *P. fragrans* as the major class of compounds for the first time in the species (Fig. 6, clusters J & K). For instance, cluster J comprises most of the gallic acid derivatives that may share the two main fragments of gallic acid (*m/z* 125.0237 and 169.0135) or the neutral loss of the galloyl moiety (152 amu). For case, peaks 27 and 42 were tentatively identified as mucic acid lactone-*O*-gallate (*m/z* 343.0684 [M–H]^−^, C_13_H_11_O_11_^−^) and mucic acid lactone-*O*-digallate (*m/z* 495.0878 [M–H]^−^, C_20_H_15_O_15_^−^), respectively, with an edge mass difference of 152 amu (galloyl moiety, C_7_H_4_O_4_^−^)^54^.

Notably, two prodelphinidin dimers consisting of epi/gallocatechin-epi/gallocatechin (*m/z* 609.1269 [M–H]^−^, peak 30, C_30_H_25_O_14_^−^) and epi/gallocatechin-epi/catechin (*m/z* 593.1312 [M–H]^−^, peak 35, C_30_H_25_O_13_^−^) were tentatively identified in the negative mode as single nodes in the GNPS molecular network (Fig. 6)^55^.

### Coumarins

Coumarins are well known for their diverse pharmacological properties such as being antioxidant, anti-inflammatory, neuroprotective, and anti-cancer^56,57^. Coumarins of the benzopyrone type were predominant in the three species for the first time (expressed as single nodes in GNPS molecular networks), with only hydroxy dimethoxy coumarin (umckalin) and hydroxy coumarin (umbelliferone) that have been isolated before from the acetone extract of *P. fragrans* which demonstrated significant antiparasitic, antioxidant, and antifungal activities^28^. Peaks 33, 48, 51, 71, 74, 121, 122, and 140 were tentatively identified in the negative mode as dihydroxy coumarin hexoside, dihydroxy coumarin, hydroxy coumarin, dihydroxy methoxy coumarin hexoside, dihydroxy methoxy coumarin, hydroxy methoxy coumarin, methoxy coumarin, and hydroxy dimethoxy coumarin, respectively^58^. In accordance, fraxetin, fraxetin-7-β-glucopyranoside, 7-methoxycoumarin-6-β-glucopyranoside, and umckalin were isolated before from *P. sidoides*^47^.

Fragmentation of coumarin in the positive mode (*m/z* 147.0440 [M + H]^+^, C_9_H_7_O_2_^+^) yielded daughter ions at *m/z* 129.0135 ([M + H]^+^–H_2_O), 119.0585 ([M + H)]^+^–CO^−^), and 103.0446 ([M + H]^+^–CO_2_^−^). As well, MS^2^ data of hydroxy methoxy coumarin in the negative mode (*m/z* 191.0345 [M–H]^−^, C_10_H_7_O_4_^−^) showed daughter ions at *m/z* 173.0187 ([M–H]^−^–H_2_O), 163.0148 ([M–H]^−^–CO^−^), 161.0002 ([M–H]^−^–OCH_2_^−^), 147.0281 ([M–H]^−^–CO_2_^−^), and 129.0645 (147–H_2_O)^58^.

### Phenolic glycosides

Different phenolic glycosides in the negative mode were predominant in the three species (expressed as single nodes in the GNPS molecular network). Peaks at 25, 56, 61, and 69 were tentatively identified for the first time in the three species as leonuriside A^59^, darendoside A^60^, crosatoside B^61^, and comososide^62^, respectively.

### Fatty acids

Several hydroxy fatty acids were predominant in the three species and provisionally identified for the first time. For instance, peaks 147 and 152 were tentatively identified in the negative mode as hydroxy octadecatrienoic acid (*m/z* 293.2125 [M–H]^−^, C_18_H_29_O_3_^−^) and hydroxy octadecadienoic acid (*m/z* 295.2281 [M–H]^−^, C_18_H_31_O_3_^−^), respectively, with a mass difference of 2 amu distinguishing them^63^.

### Phospholipids

Recently, there has been a growing interest in the utilization of phospholipids in pharmaceuticals. The wide range of molecular structures found in phospholipids contributes to their diverse biological activities. Important components of phospholipids include polar head groups like choline and amino groups, and non-polar groups like *n*-3 polyunsaturated fatty acids. These elements play significant roles in our food system and overall health, particularly in terms of antioxidant activity, memory enhancement, immune system improvement, and the prevention of cardiovascular diseases^64^.

Among the annotated compounds in the negative ionization mode, only one glyceride derivative was tentatively identified. This annotation was based on the observation of the loss of the glycerol moiety (92 amu), resulting in a fragment ion at *m/z* 391.2281, which was assigned as Lysophosphatidylglycerol (154, *m/z* 483.2731 [M–H]^−^, C_22_H_44_O_9_P^−^)^65^. Additionally, cluster M in the negative ionization mode (Fig. S6, cluster M) showed the annotation of peaks 148 and 150 as Lysophosphatidylethanolamine PE (18:2) and Lysophosphatidylethanolamine PE (16:0)^66^. On the other hand, choline phospholipid metabolites were detected in the positive ionization mode (Fig. S6, cluster G), exhibiting an even mass weight. Peak 144 (*m/z* 518.3244 [M+H]^+^, C_26_H_49_NO_7_P^+^) and peak 149 (*m/z* 520.3497 [M+H]^+^, C_26_H_51_NO_7_P^+^) were provisionally identified as lysophosphatidylcholine LPC (18:3) and lysophosphatidylcholine LPC (18:2), respectively. The presence of a characteristic fragment ion at *m/z* 184.0746, which corresponds to the phosphatidylcholine cation, along with a base peak of ([M+ H]^+^–H_2_O), provided support for these annotations^67^.

### Organic acids

The profile of organic acids in the three species was characterized here for the first time. For instance, tartaric acid (3), mucic acid (4), threonic acid (5), xylonic acid (7), and gluconic acid/galactonic acid (8) were predominant in the three species and tentatively identified in the negative mode (Fig. S7, cluster L)^68^. It was previously reported by Williams and Harborne^53^ that tartaric acid is a characteristic constituent found in the genus *Pelargonium*.

On the other hand, dicarboxylic acids (expressed as single nodes in the GNPS molecular network) were predominant in the three species. For case, peaks 67, 97, 115, and 123 were provisionally identified (in the negative mode) as pimelic acid, suberic acid, azelaic acid, and sebacic acid, respectively. The MS^2^ data of each was mainly characterized by the loss of 2CO^−^; sebacic acid (*m/z* 201.1128 [M–H]−, C_10_H_17_O_4_^−^) fragmentation led to the daughter ions *m/z* 183.0298 ([M–H]^−^–H_2_O), 157.1486 ([M–H]^−^–CO_2_^−^), and 139.1281 (183–CO_2_^−^)^69^.

### Data processing and multivariate data analyses

Multivariate data analysis techniques such as principal component analysis (PCA), hierarchical cluster analysis (HCA), and Partial Least Squares Discriminant Analysis (PLS-DA) were utilized to investigate the relative variability between the selected three *Pelargonium* species. These analyses allowed for the classification of samples into distinct groups based on their metabolite distribution^70^. Both HCA and PCA are unsupervised methods commonly used for assessing natural inter-relationships, including grouping, clustering, and identifying outliers in datasets without prior knowledge. The PCA score plots using PC1/PC2 (Fig. S8, A) revealed that *P. fragrans* was positioned far away from the other two species, indicating its distinct metabolite profile. These findings were validated through the application HCA, another unsupervised multivariate data analysis technique used to explore variations among different LC–MS samples. HCA, an excellent preliminary data analysis tool, grouped the extracts into two main clusters based on their chemical profiles (Fig. S8, B). Cluster I included *P. graveolens* and *P. denticulatum;* the small distance between the two samples within each cluster indicated high similarity in their chemical profiles. Cluster II solely comprised *P. fragrans*, indicating significant differences in its metabolic profile compared to the other cluster.

**Fig. 7:**
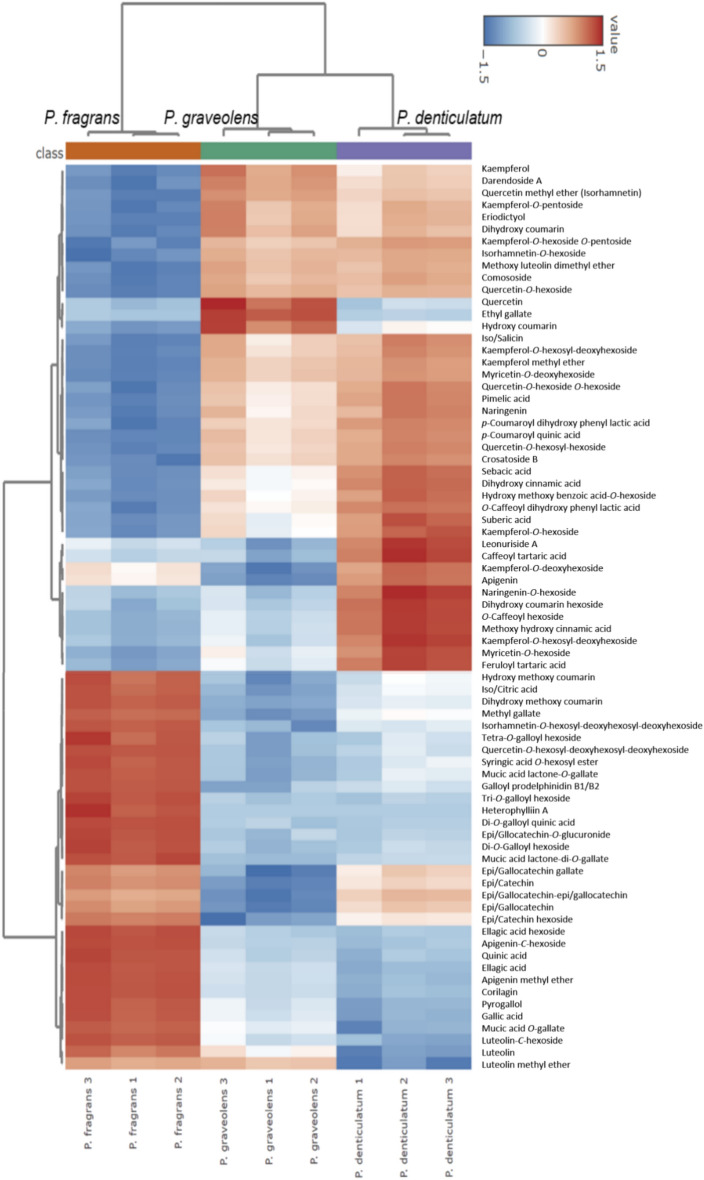
Heat map of relative abundance of compounds annotated from the three *Pelargonium* species. Y axis represents the relative abundance (log-transformed and Pareto-scaled peak areas of ion counts for each annotated metabolite), while X axis represents the values of the three replicates that were used for each species. The relative abundances of the same analyte across all samples in both data sets (positive or negative ESI condition) were selected based on the mode in which the analyte exhibited higher sensitivity and was used for representation. The color scale represents the log-scaled values of metabolite abundance. Increased and decreased metabolite levels in tested samples are represented by red and blue, respectively. For hierarchical clustering in heat map, Euclidean distances were used for distance measures and Ward’s linkage was used for clustering algorithms.

Heat maps were generated to visualize the distribution of metabolites in the different species by assigning color intensity through a scale. In this scale, blue indicates down-regulated metabolites, while red indicates up-regulated metabolites (Fig. 7). Interestingly, the three samples contained a majority of the annotated metabolites from the various classes of compounds. Notably, *P. graveolens* and *P. denticulatum* exhibited a higher abundance of flavonol mono- and di-*O*-glycosides along with cinnamic acid derivatives compared to *P. fragrans* which was rich in tannins and flavone *C-*glycosides, which were less predominant in *P. denticulatum* and *P. graveolens*. On the other hand, coumarins were predominant in all three species. The results of the generated heat map align perfectly with the GNPS molecular networking in relation to the predominance of the different compounds in the three species.

**Fig. 8:**
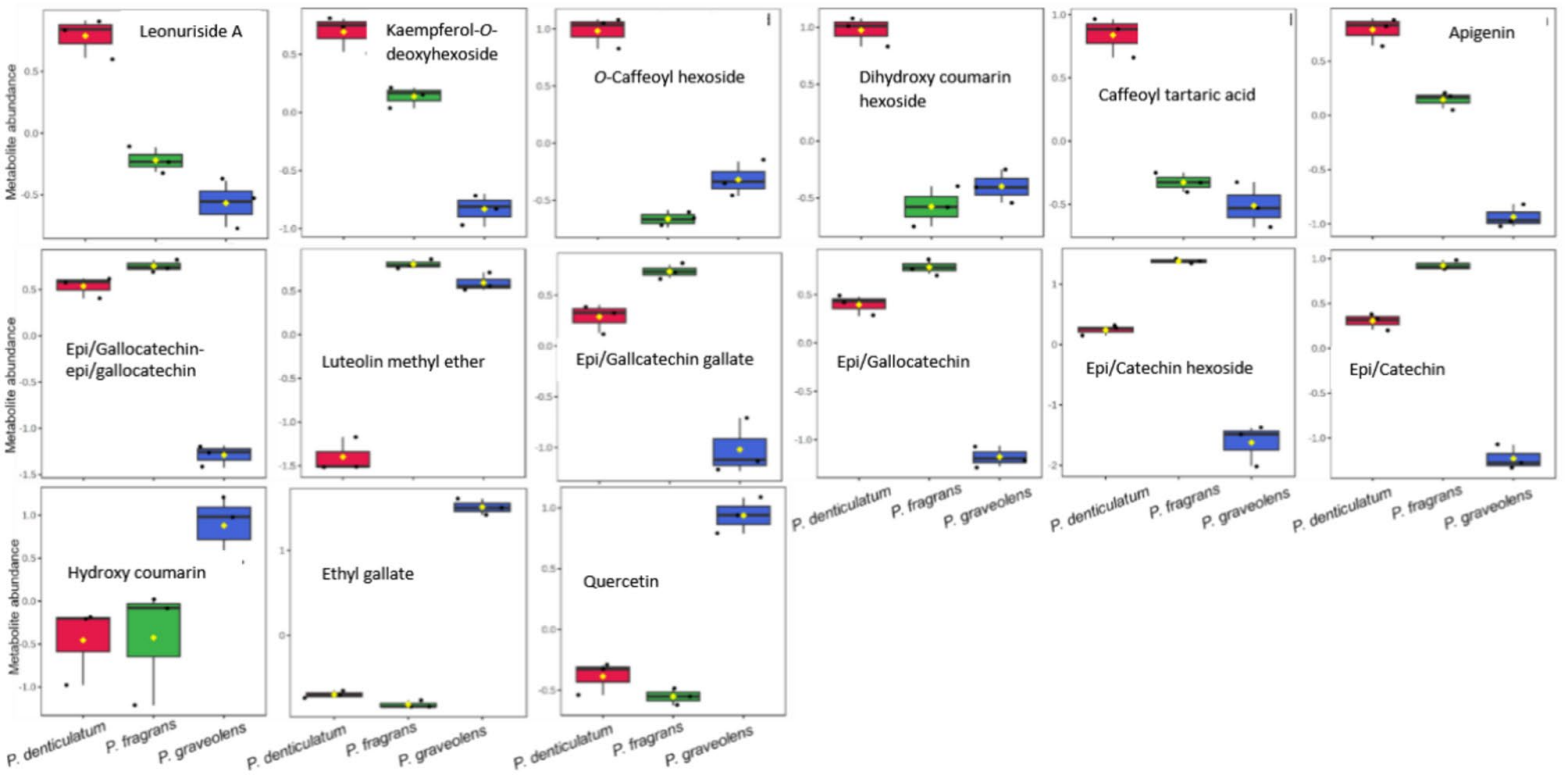
Relative abundance of metabolites annotated from the three *Pelargonium* species. The box and whisker plots summarize the metabolite values. Y axes represent relative units (log-transformed and Pareto-scaled data). X axes represent the three *Pelargonium* species. Single data points are indicated by circles, while medians are indicated by horizontal lines.

On the other hand, PLS-DA, a supervised multivariate data analysis technique that considers sample labels, was used to achieve dimensionality reduction. It successfully grouped the samples based on their chemical profiles, with *P. fragrans* being the most distinct (Fig. S8, C). Relevant variables highlighting the differences among the three species were represented using the Variable Importance in Projection (VIP) plot of the PLS-DA model (Fig. S8, D). As well, box and whisker plots summarized the marker compounds of the three species (Fig. 8); tannins such as epi/gallocatechin gallate and epi/gallocatechin-epi/gallocatechin were considered as marker compounds for *P. fragrans.* Further, phenolic acids such as *O*-caffeoyl hexoside and caffeoyl tartaric acid, along with the flavonoids; kaempferol-*O-*deoxyhexoside and apigenin, were considered as marker compounds in *P. denticulatum*. On the other hand, quercetin, ethyl gallate, and hydroxy coumarin were characterized as marker compounds in *P. graveolens.*

Thus, our study offered a thorough analysis of the metabolic profiles of *Pelargonium graveolens, Pelargonium denticulatum,* and *Pelargonium fragrans*, cultivated in Egypt. The exploration of the diverse metabolites and their interconnections carries significant implications for recognizing the significance of bioactive-rich *Pelargonium* species in the realms of drug discovery.

## Conclusion

To the best of our knowledge, this is the first comprehensive report on the metabolic profiles of three *Pelargonium* species cultivated in Egypt using UPLC-MS/MS, GNPS molecular networking, and multivariate analysis, providing satisfactory discrimination between them. The study emphasizes the significant presence of tannins and flavone *C*-glycosides in *P. fragrans*, while *P. denticulatum* and *P. graveolens* exhibit a notable abundance of flavonol mono- and di-*O*-glycosides, and cinnamic acid derivatives. Coumarins and benzoic acid derivatives are prevalent in the three species, along with organic acids, fatty acids, and phospholipids. Uncovering the diverse metabolites and their interconnections holds great importance in understanding the importance of bioactive-rich *Pelargonium* species in the domains of drug discovery and disease targeting.

## Supplementary Information

Supplementary Information 1.

Supplementary Figures.

## Data Availability

The manuscript contains all the data that support the findings.

## Abbreviations

GC–MS: Gas chromatography-mass spectrometry
LC–MS: Liquid chromatography-mass spectrometry
GNPS: Global natural product social
cAMP: Cyclic adenosine monophosphate
PCA: Principal component analysis
HCA: Hierarchical cluster analysis
PLS-DA: Partial least squares discriminant analysis
