# Supplementary material for: A comparative UPLC-orbitrap-MS-based metabolite profiling of three Pelargonium species cultivated in Egypt
Source: Sci Rep. 2024 Oct 1;14:22765. doi: 10.1038/s41598-024-72153-0 (PMC11445532; doi:10.1038/s41598-024-72153-0)
Supplement: Supplementary file 2 — Supplementary Figures. [file 41598_2024_72153_MOESM2_ESM.docx]

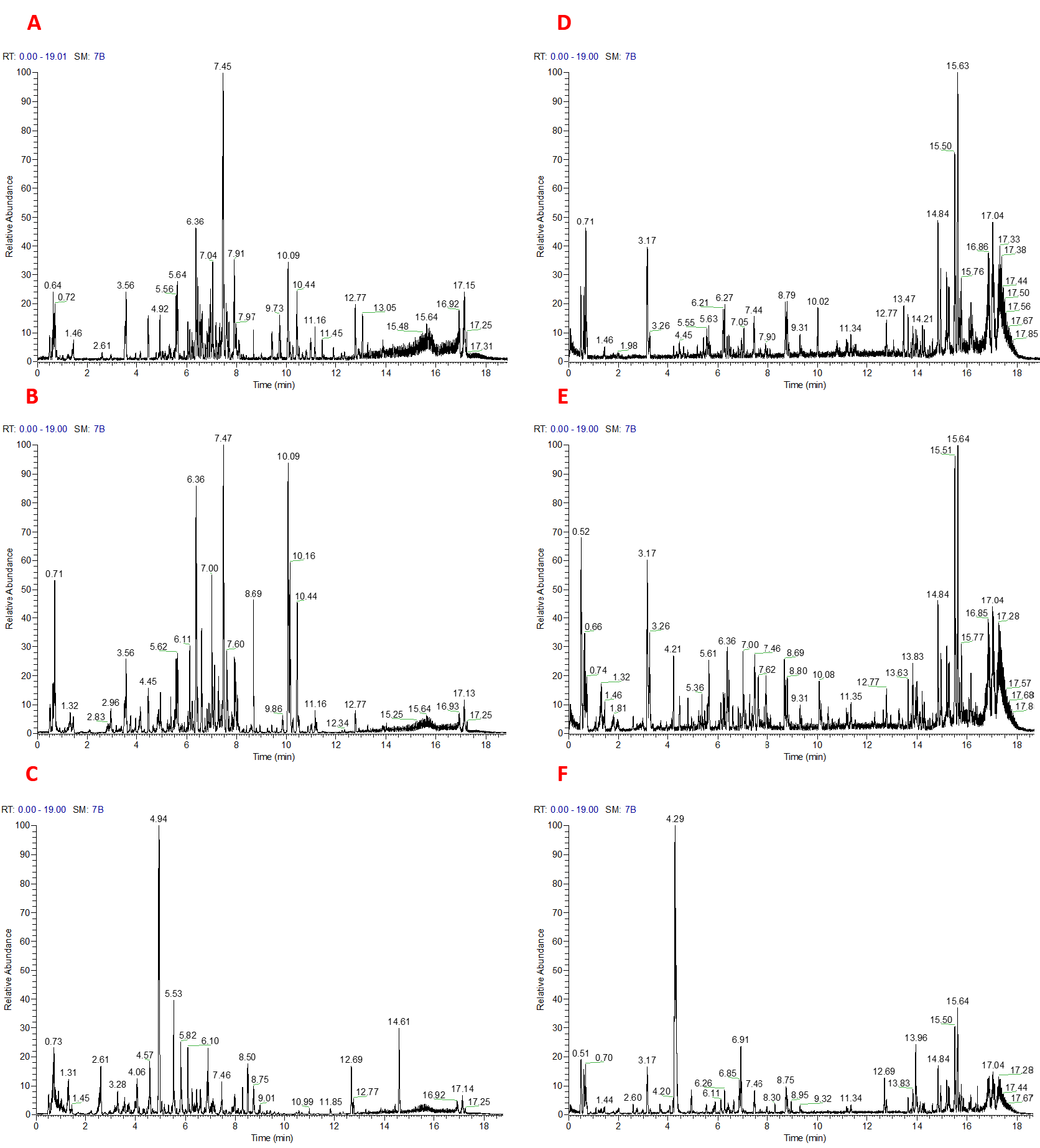


**Fig. S1.** The base peak ion chromatograms in both negative (A, B, and C) and positive (D, E, and F) ionization modes of *P. graveolens, P. denticulatum, and P. fragrans,* respectively.

**
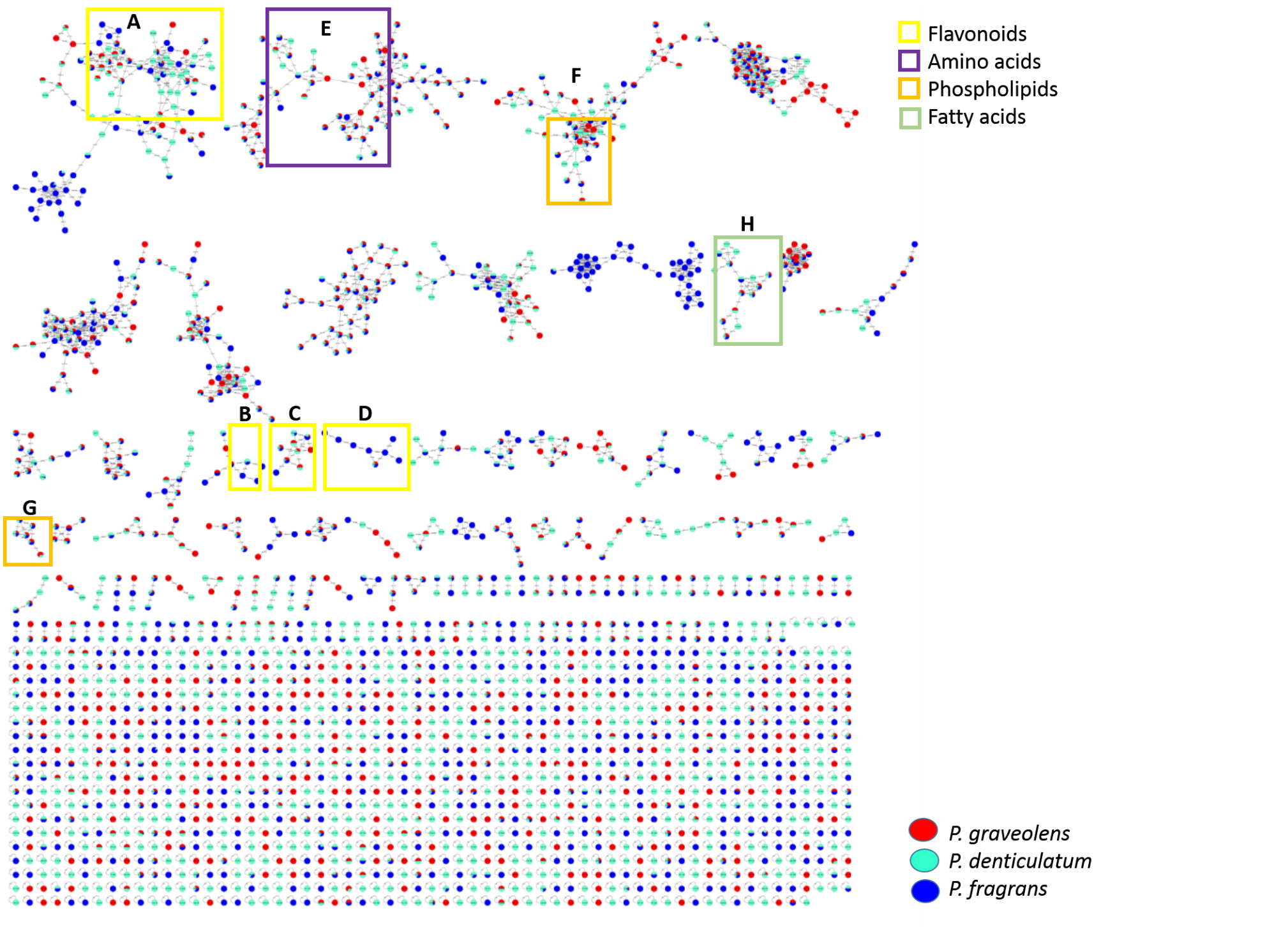
**

**Fig. S2.** GNPS molecular networking performed using MS/MS data in positive mode. The selected clusters indicate the different classes of the annotated metabolites. A: Flavonol mono- and di-*O*-glycosides. B: Flavan-3-ols. C: Methylated flavonoids. D: Flavone *C*-glycosides. E: Amino acids. F & G: Phospholipids. H: Fatty acids. Pie charts were used to depict the distribution of ions in *P. graveolens, P. denticulatum,* and *P. fragrans*.

**
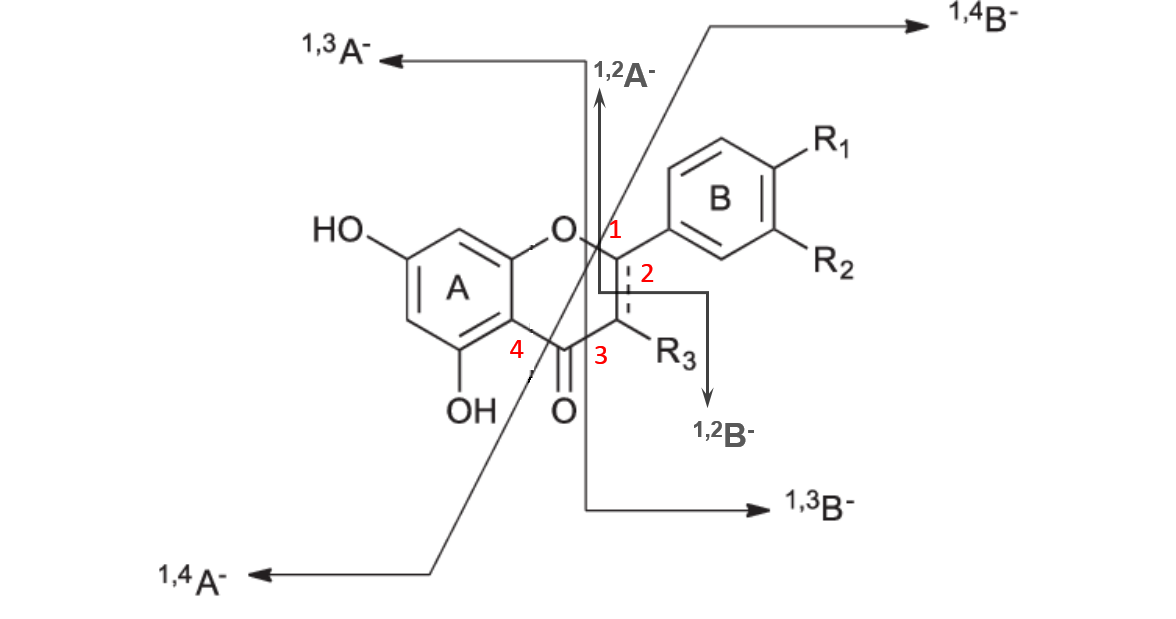
**

**Fig. S3.** Nomenclature for the RDA fragments adopted for different classes of flavonoids. Bonds of concern are numbered from 1 to 4.

*
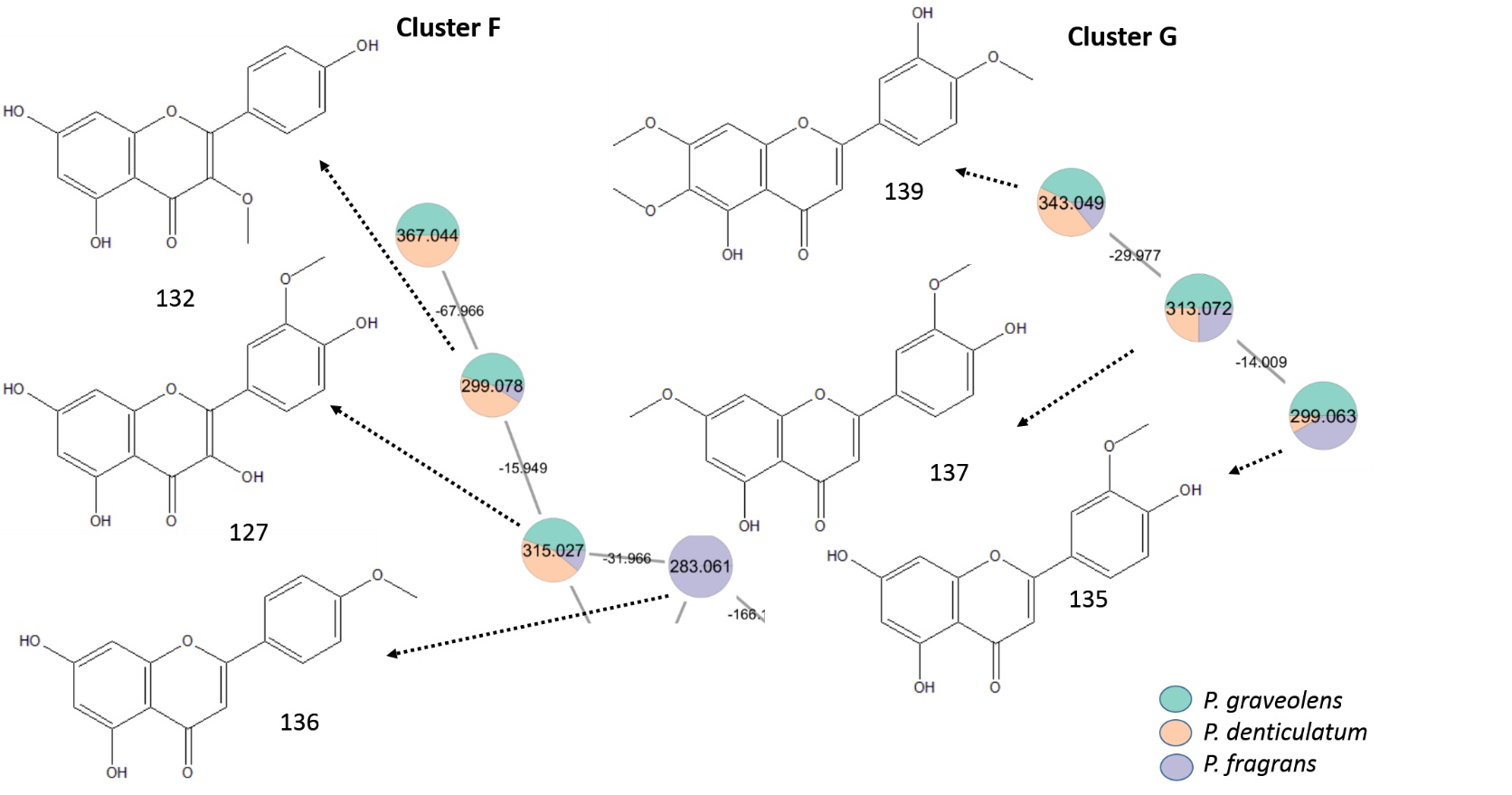
*

**Fig. S4.** Annotated methylated flavonoids in the negative mode (position of substitutions may vary) and their distribution in the GNPS molecular network from *P. graveolens, P. denticulatum,* and *P. fragrans.*

*
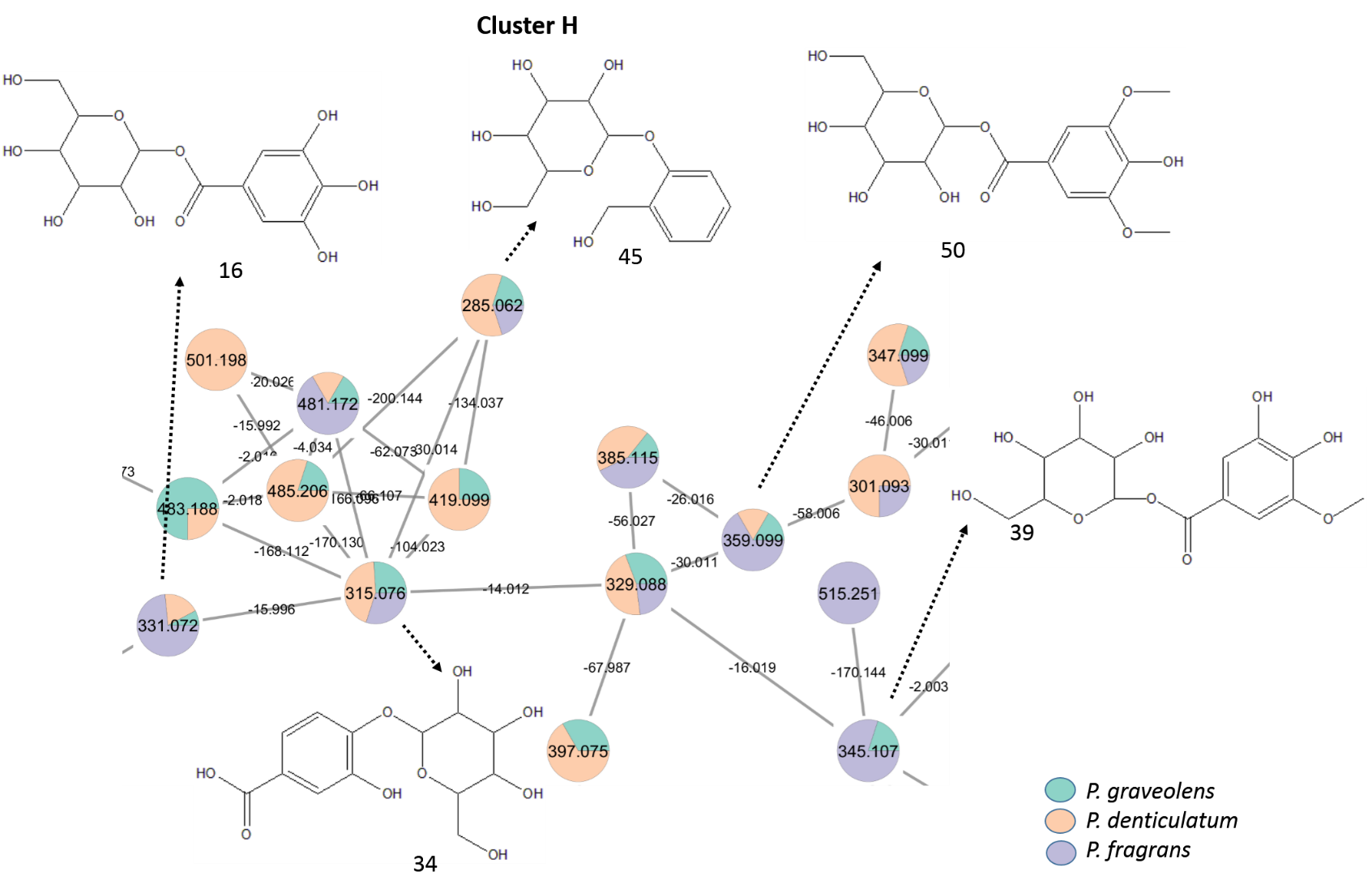
*

**Fig. S5.** Annotated phenolic acids (benzoic acid deriv.) in the negative mode (position of substitutions may vary) and their distribution in the GNPS molecular network from *P. graveolens, P. denticulatum,* and *P. fragrans.*

*
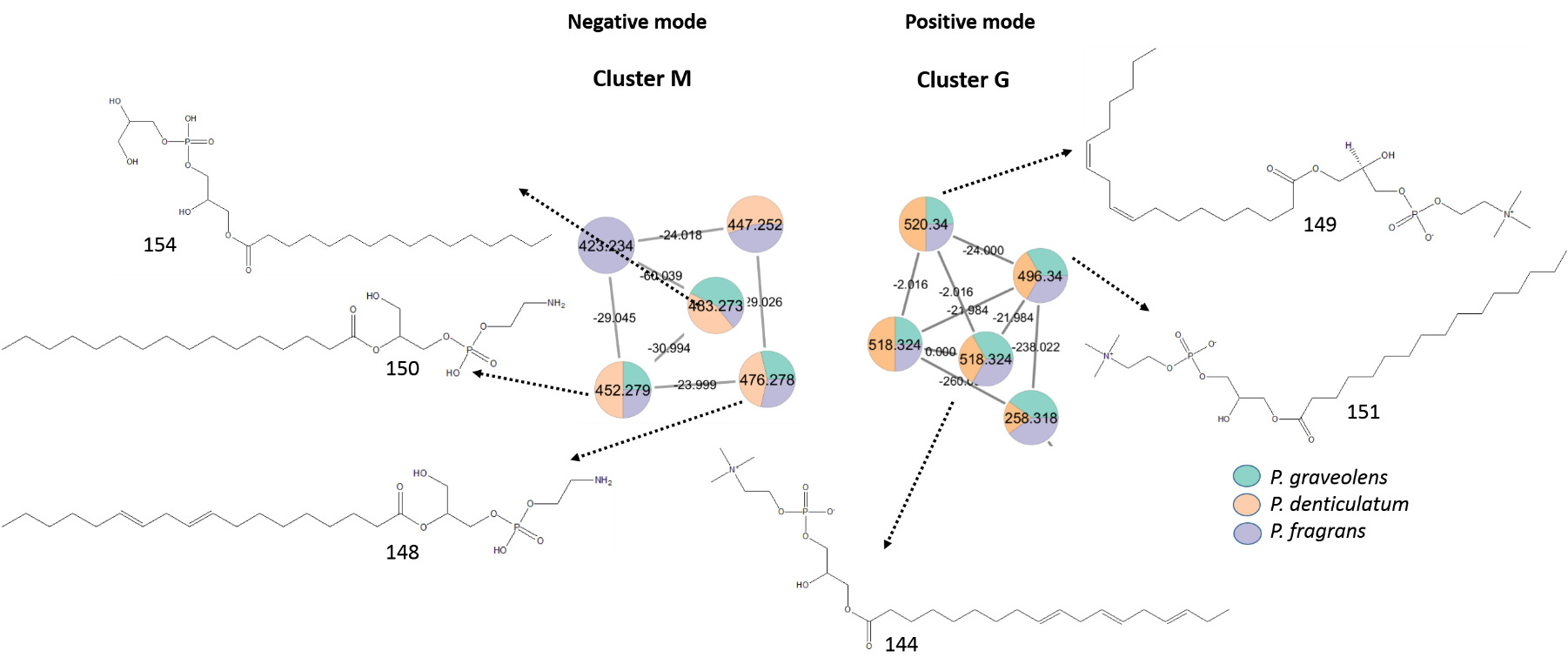
*

**Fig. S6.** Annotated phospholipids in the negative and positive modes (position of substitutions may vary) with their distribution in the GNPS molecular network from *P. graveolens, P. denticulatum,* and *P. fragrans.*

*
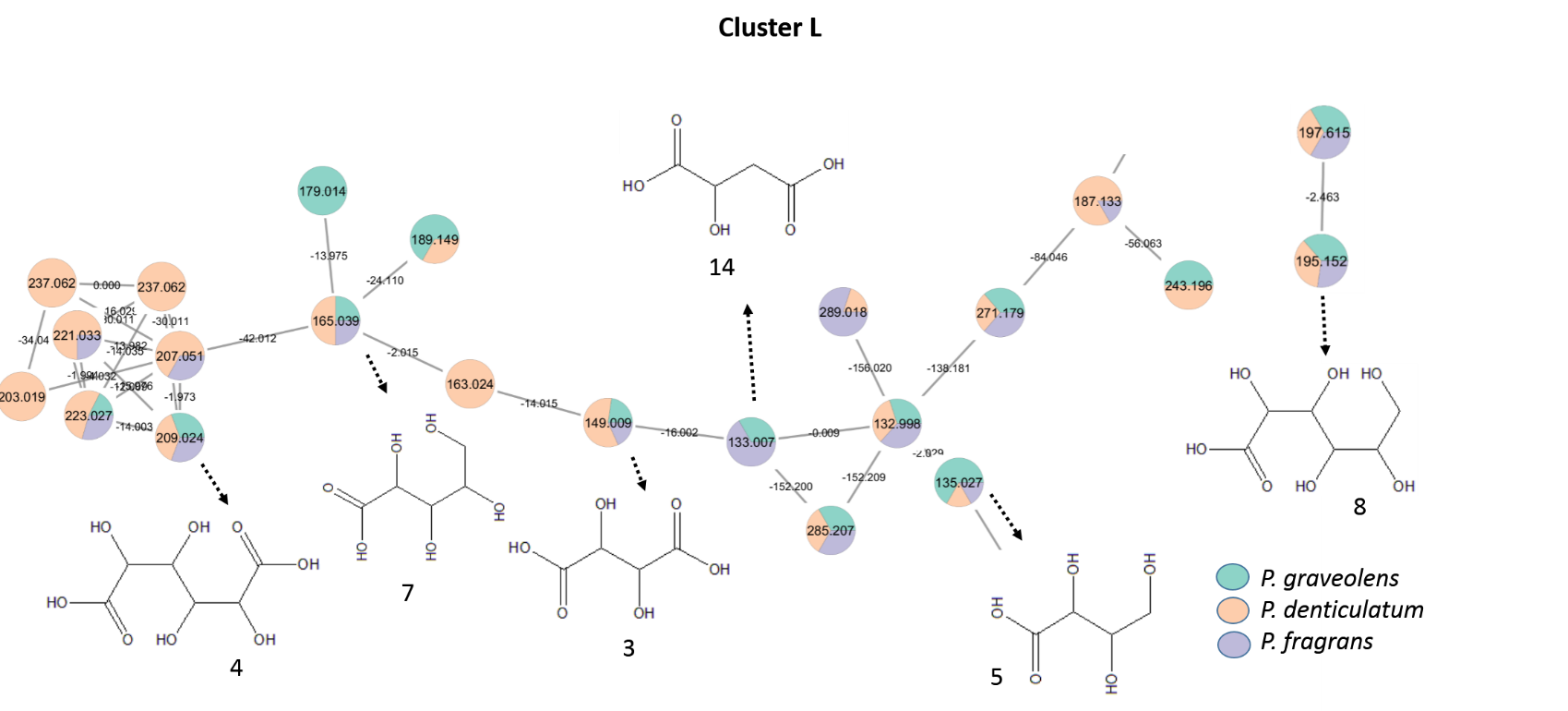
*

**Fig. S7.** Annotated organic acids in the negative mode (position of substitutions may vary) and their distribution in the GNPS molecular network from *P. graveolens, P. denticulatum,* and *P. fragrans.*


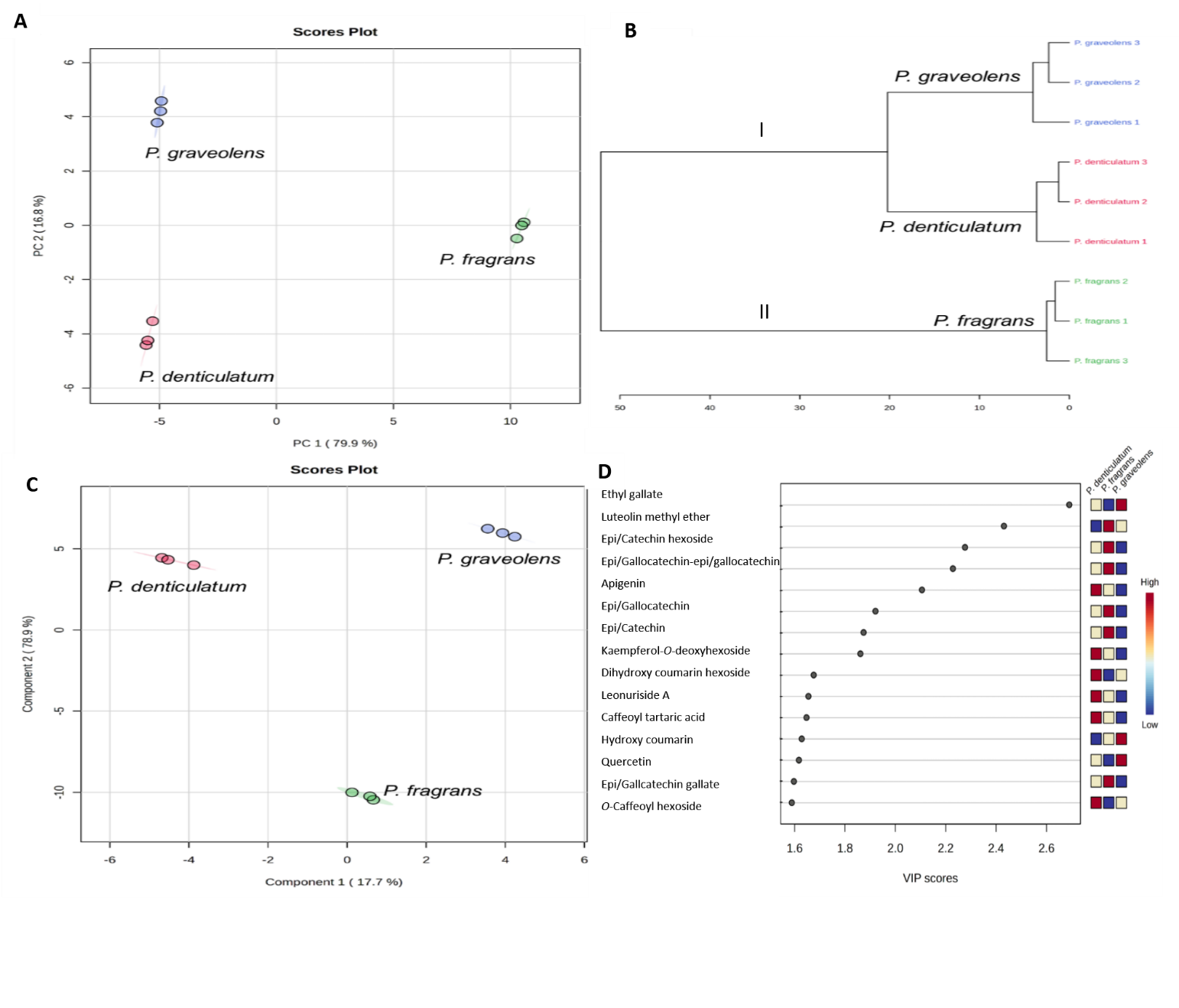


**Fig. S8.** Two-dimensional Principal Component Analysis (PCA) score plot (A), Hierarchical Cluster Analysis (HCA) (B), Partial Least Squares Discriminant Analysis (PLS-DA) score plot (C), and Variable Importance in Projection (VIP) plot of the PLS-DA model (D) of metabolites annotated from the three *Pelargonium* species.
